# Novel quinolone chalcones targeting colchicine-binding pocket kill multidrug-resistant cancer cells by inhibiting tubulin activity and MRP1 function

**DOI:** 10.1038/s41598-017-10972-0

**Published:** 2017-08-31

**Authors:** I. Kalhari Lindamulage, Hai-Yen Vu, Chandrabose Karthikeyan, James Knockleby, Yi-Fang Lee, Piyush Trivedi, Hoyun Lee

**Affiliations:** 1 Health Sciences North Research Institute, 41 Ramsey Lake Road, Sudbury, Ontario, P3E 5J1 Canada; 2 Biomolecular Sciences, Laurentian University, 935 Ramsey Lake Road, Sudbury, Ontario, P3E 2C6 Canada; 3 Departments of Medicine, the Faculty of Medicine, the University of Ottawa, Ottawa, Ontario, K1H 5M8 Canada; 4 School of Pharmaceutical Sciences, Rajiv Gandhi Technical University, Airport Bypass Rd, Gandhi Nagar, Bhopal, M.P India

## Abstract

Agents targeting colchicine-binding pocket usually show a minimal drug-resistance issue, albeit often associated with high toxicity. Chalcone-based compounds, which may bind to colchicine-binding site, are found in many edible fruits, suggesting that they can be effective drugs with less toxicity. Therefore, we synthesized and examined 24 quinolone chalcone compounds, from which we identified ((E)-3-(3-(2-Methoxyphenyl)-3-oxoprop-1-enyl) quinolin-2(1H)-one) (CTR-17) and ((E)-6-Methoxy-3-(3-(2-methoxyphenyl)-3-oxoprop-1-enyl) quinolin-2(1H)-one) (CTR-20) as promising leads. In particular, CTR-20 was effective against 65 different cancer cell lines originated from 12 different tissues, largely in a cancer cell-specific manner. We found that both CTR-17 and CTR-20 reversibly bind to the colchicine-binding pocket on β-tubulin. Interestingly however, both the CTRs were highly effective against multidrug-resistant cancer cells while colchicine, paclitaxel and vinblastine were not. Our study with CTR-20 showed that it overcomes multidrug-resistance through its ability to impede MRP1 function while maintaining strong inhibition against microtubule activity. Data from mice engrafted with the MDA-MB-231 triple-negative breast cancer cells showed that both CTR-17 and CTR-20 possess strong anticancer activity, alone or in combination with paclitaxel, without causing any notable side effects. Together, our data demonstrates that both the CTRs can be effective and safe drugs against many different cancers, especially against multidrug-resistant tumors.

## Introduction

The microtubule cytoskeleton is a well-validated cancer therapeutic target^1^. There are at least four binding sites on tubulin that can disrupt microtubule dynamics: taxanes, vinca alkaloids, laulimalide and colchicine^2–4^. Tubulin inhibitors targeting the first two sites such as paclitaxel (Tax) and vinblastine are widely used to treat many different cancers^5–8^. However, they often show dose-limiting toxicity and face a multidrug drug-resistance (MDR) issue^9–13^, usually due to the high expression of p-glycoprotein (p-gp; MDR1) or multidrug resistance-associate proteins (MRPs)^9, 14^. The overexpression of β-tubulin isoforms and certain mutations also render resistance to taxanes^14, 15^. Unlike taxanes and vinca alkaloids, agents targeting colchicine-binding site have a minimal multidrug resistance issue, and can also overcome the overexpression of β-tubulin isoforms^1, 16, 17^. However, a drawback is that colchicine and its derivatives are also very toxic to humans^1, 4^. Therefore, developing a microtubule inhibitor that binds to the colchicine-binding site with low side effects can be highly desirable^4, 5, 18, 19^.

**Figure 1:**
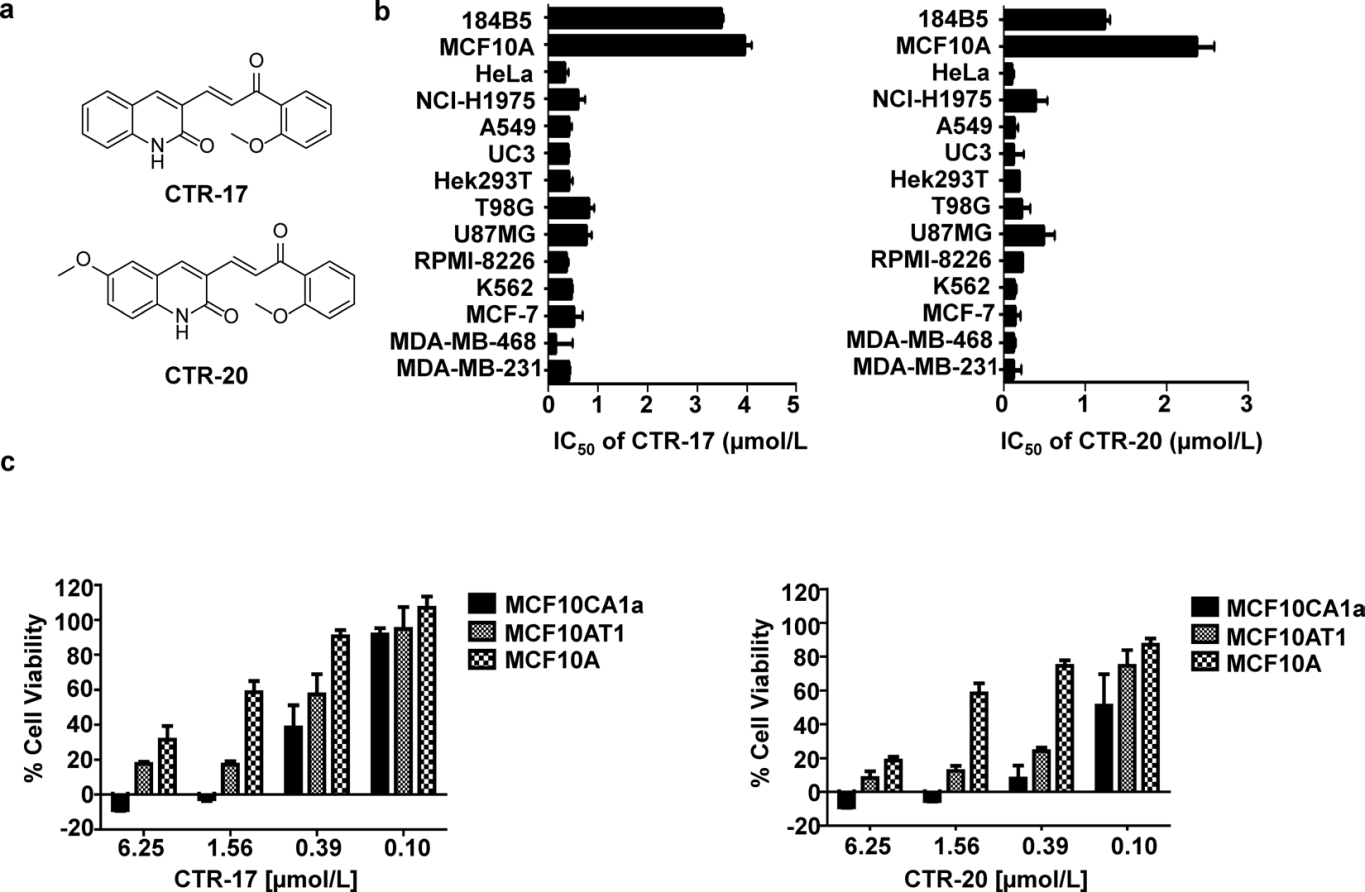
Cancer cells are more sensitive to CTRs than non-cancerous cells. (**a**) Chemical structures of (E)-3-(3-(2-Methoxyphenyl)-3-oxoprop-1-enyl)quinolin-2(1H)-one (CTR-17) and (E)-6-Methoxy-3-(3-(2-methoxyphenyl)-3-oxoprop-1-enyl) quinolin-2(1H)-one (CTR-20). (**b**) CTR-17 and CTR-20 preferentially kill many different cancer cells over non-cancer cells (184B5 and MCF 10A). (**c**) Both CTR-17 and CTR-20 preferentially kill/inhibit proliferation of fully malignant breast cancer cells (MCF 10CA1a) over isogenic premalignant (MCF 10AT1) or non-cancer breast (MCF 10A) cells.

With a central core composed of an aromatic ketone and an enone group, chalcone-based compounds have been reported to show potent anti-tubulin activity^4^. Since the binding of certain chalcones to tubulin can be inhibited by colchicine, they may directly bind to β-tubulin through the colchicine-binding pocket^20–24^. In addition, chalcones are not only used to treat many different diseases such as ulcers and skin disorders, but also abundantly found in many edible fruits. This may suggest that chalcones can be relatively safe to humans^25^. In agreement, we found previously that certain chalcone derivatives preferentially kill cancer over non-cancer cells^26^. Therefore, we synthesized and examined 24 novel chalcone-derivatives, among which CTR-17 and CTR-20 (Fig. 1a) were identified as highly promising leads as they effectively and preferentially killed cancer over non-cancer cells. Both the CTR compounds bind to the colchicine binding pocket and cause a prolonged mitotic arrest at the spindle assembly checkpoint (SAC), eventually leading to cell death. Importantly, both CTR-17 and CTR-20 effectively killed MDR1- and MRP1-overexpressing tumor cells that showed resistance to colchicine, paclitaxel and other agents. Furthermore, when used in combination with paclitaxel or ABT-737 (Bcl2 family protein inhibitor), the CTR compounds showed strong synergistic effects against tumor cells, including multidrug-resistant tumors. Finally, both CTR-17 and CTR-20 showed strong anti-tumor activity in mice engrafted with metastatic breast cancer cells, without showing any notable side effects.

## Results

### CTR-17 and CTR-20 preferentially kill a wide range of malignant cells over non-cancer cells

Initial screening of the CTR library using three breast cancer cell lines (MCF-7, MDA-MB-468 and MDA-MB-231) and two non-cancer breast cell lines (MCF 10A and 184B5) identified CTR-17 ((E)-3-(3-(2-Methoxyphenyl)-3-oxoprop-1-enyl) quinolin-2(1H)-one) and CTR-20 ((E)-6-Methoxy-3-(3-(2-methoxyphenyl)-3-oxoprop-1-enyl) quinolin-2(1H)-one) (Fig. 1a) as promising lead compounds since they effectively and preferentially killed the three cancer cell lines over the two non-cancer cell lines. (The detailed information on the design, synthesis, characterization, and biological effects of the 24 novel chemicals were described in our patent application published recently [WO 2017/083979, 2017], and will be reported in an appropriate scientific journal in near future). Data obtained from our subsequent works showed that both CTR-17 and CTR-20 effectively killed a wide range of different cancer cell lines including cancers originated from cervix, lung, bladder, kidney, brain, multiple myeloma, lymphoma and breast, at IC_50_ values from 0.12 μmol/L (MDA-MB-468 by CTR-20) to 1.11 μmol/L (U87MG by CTR-20) (Fig. 1b). Importantly, both CTR-17 and CTR-20 killed cancer cells 20–26 folds more effectively than non-cancerous cells (MCF 10A and 184B5) (Fig. 1b). We then examined the efficacy of CTR-20 against the NCI-60 cancer panel in collaboration with the US National Cancer Institute. As shown in Supplementary Figs S1–S5, CTR-20 effectively killed/inhibited proliferation of all the cell lines included in the NCI-60 panel.

To gain further insights into their preferential killing of cancer over non-cancer cells, we examined the cytotoxic effects of CTR-17 and CTR-20 against three MCF 10A isogenic cell lines. As shown in Fig. 1c, both CTR-17 and CTR-20 preferentially killed the fully malignant MCF 10CA1a breast cancer cells over the premalignant MCF 10AT1 and non-cancer MCF 10A breast cells. For example, the cell survival rates at 0.39 μmol/L of CTR-20 were 10%, 20% and 75%, respectively, for MCF 10AC1a, MCF 10AT1 and MCF 10A. This data is also consistent with the observation that CTR-17 caused PARP cleavage in HeLa cells but not in 184B5 (Supplementary Fig. S6).

### CTR-17 and CTR-20 effectively kill cancer cells overexpressing MDR1 or MRP1, synergistically when combined with paclitaxel

**Figure 2:**
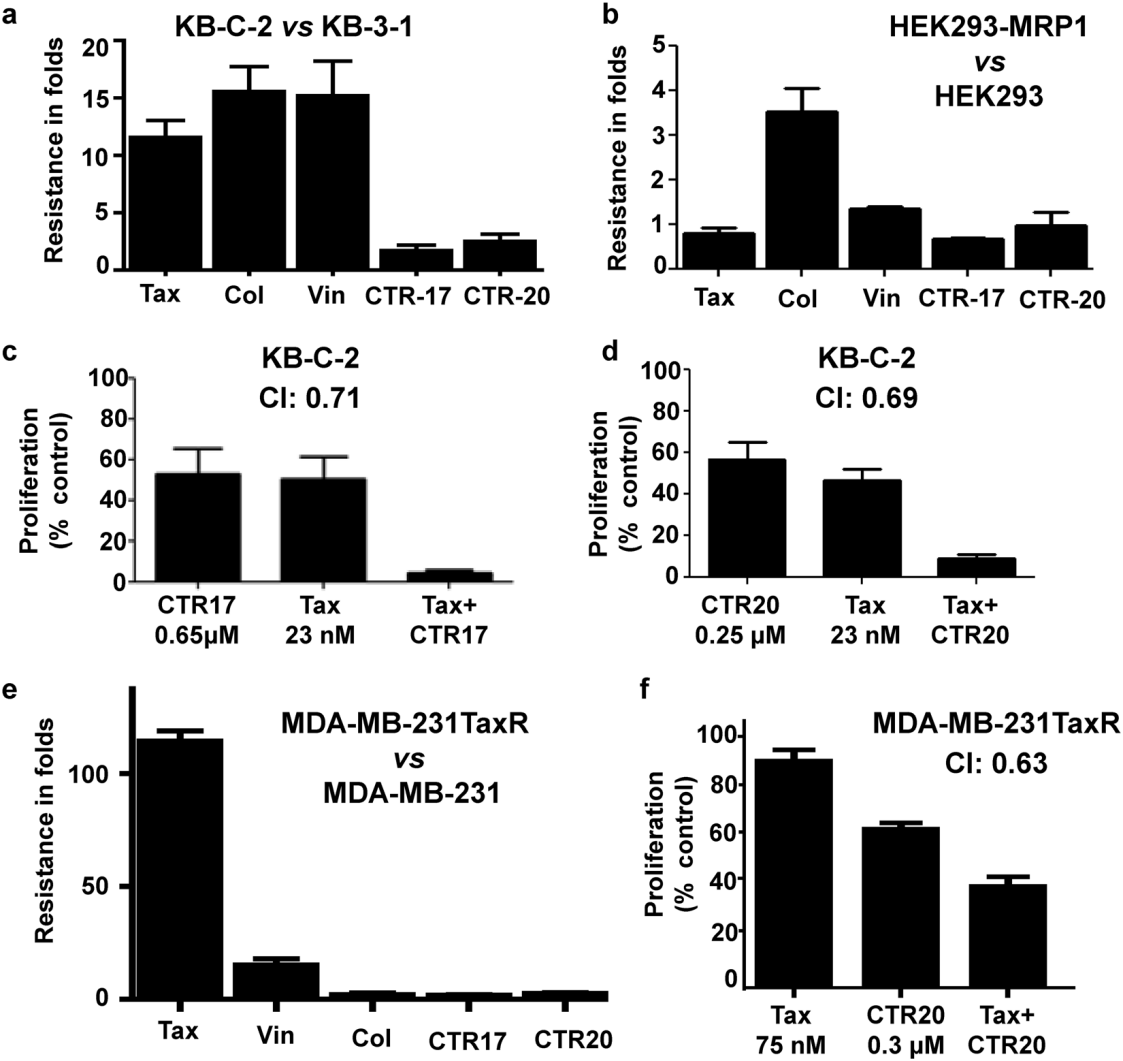
CTRs effectively killed multidrug-resistant cancer cells, synergistically when combined with paclitaxel. (**a**,**b**) CTR-17 and CTR-20 effectively kill cells overexpressing MDR1 (**a**) or MRP1 (**b**). Note that the multidrug-resistant KB-C-2 and HEK293-MRP1 cells were developed from KB-3-1 cervical cancer cell line and HEK293 kidney cell line, respectively. Tax, Col and Vin denote paclitaxel, colchicine and vinblastine, respectively. (**c,d**) When combined with Tax, CTRs showed substantial synergistic cell-killing effects against KB-C-2 cells. The same concentrations were used for both single and combinational treatments. Combinational index (CI) < 1.0, CI = 1.0 and CI > 1.0 are synergistic, additive and antagonistic, respectively^27^. (**e**) Both CTR-17 and CTR-20 effectively killed multidrug (paclitaxel)-resistant MDA-MB-231TaxR. The MDA-MB-231TaxR, which is 114 times more resistant to paclitaxel, was developed in house by culturing the MDA-MB-231 triple-negative metastatic breast cancer cell line in incrementally increased paclitaxel concentrations for over one-year period. (**f**) The combination of Tax and CTR-20 showed a strong synergistic effect against MDA-MB-231TaxR. The expression of MDR1 or MRP1 proteins in these cell lines are shown in Supplementary Fig. S7. Data presented are mean ± S.E.M value of triplicates of at least four independent experiments.

Since cancer cells overexpressing MDR1 or MRP1 are often resistant to many different chemotherapeutic agents, we examined if they are also resistant to CTRs. This idea was stemmed from the observation that CTR-20, unexpectedly, killed the temozolomide-resistant T98G better than the temozolomide-sensitive U87MG brain cancer cells (Fig. 1b). We found that IC_50_ values of CTR-17 and CTR-20 for the KB-C-2 MDR1-overexpressing cells were 0.65 ± 0.16 μmol/L and 0.25 ± 0.03 μmol/L, respectively, which are only slightly higher than their IC_50_ values against the parental KB-C-1 (0.38 ± 0.07 μmol/L for CTR-17 and 0.10 ± 0.01 μmol/L for CTR-20) (Fig. 2a; Supplementary Fig. S7a,b). This is a stark contrast with the observation that the resistance ratios of KB-C-2 *vs* KB-C-1 cells by paclitaxel (Tax), colchicine (Col) and vinblastine (Vin) were 11.48, 15.57 and 15.20 folds, respectively (Fig. 2a). We found that the MRP1-overexpressing HEK293 cells were sensitive to both CTR-17 and CTR-20, but approximately 3.5-fold more resistant to colchicine (Fig. 2b). These data are surprising given that colchicine and CTRs bind to the same binding pocket on the tubulin (see below). Another interesting point is that both CTR-17 and CTR-20 killed the H69AR multidrug-resistant small cell lung cancer cells much more effectively than the non-multidrug-resistant SW-1271 small cell lung cancer cells, as IC_50_ values of CTR-17 on SW-1271 and H69AR were 1.14 ± 0.04 μmol/L and 0.52 ± 0.10 μmol/L, respectively; and those for CTR-20 were 1.95 ± 0.01 μmol/L and 0.13 ± 0.01 μmol/L, respectively (Supplementary Table S1).

We noted that both CTR-17 and CTR-20 showed synergistic effects against multidrug-resistant KB-C-2 cells when combined with paclitaxel (Fig. 2c and d): the combinational index (CI) was 0.71 for the combination of 0.65 μmol/L CTR-17 and 23 nmol/L of paclitaxel, or 0.69 for the combination of 0.25 μmol/L CTR-20 and 23 nmol/L paclitaxel. It should be noted that CI < 1.0, CI = 1.0, and CI > 1.0 are synergistic, additive and antagonistic, respectively^27^.

To gain further insights into the CTR-mediated multidrug-resistant cell-killing, we have generated the paclitaxel-resistant MDA-MB-231TaxR cell line by culturing the MDA-MB-231 triple-negative metastatic breast cancer cells in the incrementally increasing concentrations of paclitaxel. The resultant MDA-MB-231TaxR overexpresses MDR1 (Supplementary Fig. S7c) and is 114 times more resistant to paclitaxel (Fig. 2e). However, MDA-MB-231TaxR is very sensitive to CTR-17 and CTR-20 (Fig. 2e). Moreover, the combination of paclitaxel and CTR-20 showed strong synergy (CI = 0.63) against MDA-MB-231TaxR (Fig. 2f). Since paclitaxel is quite toxic to human at clinically relevant doses, the synergic effects of paclitaxel-CTR combinations at low concentrations against multidrug-resistant cancer cells can provide new opportunities of controlling drug-resistant cancers with relatively low toxicity.

### Combination of CTR-20 with ABT-737 showed a very strong synergistic effect

**Figure 3:**
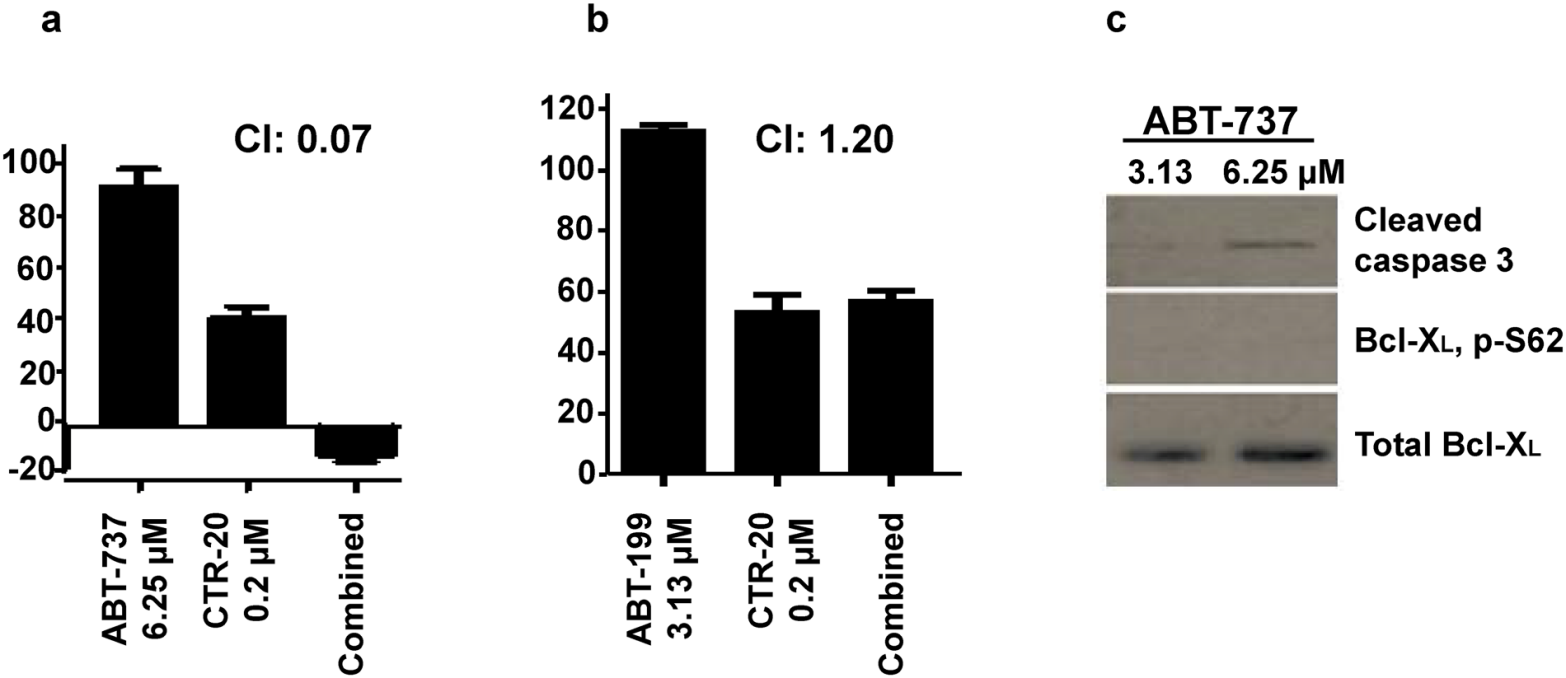
The combination of CTR-20 with ABT-737 (**a**), but not with ABT-199 (**b**), showed strong synergistic effects. MDA-MB-231 cells were treated with ABT-737 (6.25 μM), ABT-199 (3.13 μM), or combination with 0.2 μM CTR-20. “CI” denotes combinational index. IC_50_ values of ABT-373 and ABT-199 were 28.54 ± 3.99 μM and 11.58 ± 1.13 μM, respectively. (**c**) 6.25 μM ABT-737 caused the cleavage of caspase 3, but did not phosphorylate Bcl-X_L_ on the Ser62 residue.

To gain further insight into the combinational effects of CTR compounds, we examined the cell-killing effect of CTR-20 in the presence of apoptotic pathway inhibitors. We found that the combination of CTR-20 and ABT-737 was highly synergistic as its CI was 0.07 against MDA-MB-231 (Fig. 3a). In contrast, the combination of CTR-20 and ABT-199 was slightly antagonistic as its CI was 1.20 (Fig. 3b). Since ABT-199 is an inhibitor specific to Bcl-2 while ABT-737 inhibits Bcl-2, Bcl-X_L_ and Bcl-w, the synergistic effect of CTR-20 and inhibition of the anti-apoptotic pathway may be limited to Bcl-X_L_ and Bcl-w. It should also be noted that the 6.25 μmol/L of ABT-737 activated apoptosis (manifested by caspase 3 cleavage) without activating Bcl-X_L_ (i.e., phosphorylation on the Ser62 residue) (Fig. 3c). Thus, the combination of CTR-20 with ABT-737 can also be effective against a broad range of cancers without causing thrombocytopenia^28^.

### Cancer cells treated with CTRs showed defects in the centrosome alignment and chromosome segregation, leading to prolonged cell cycle arrest at the SAC checkpoint and, eventually, cell death

**Figure 4:**
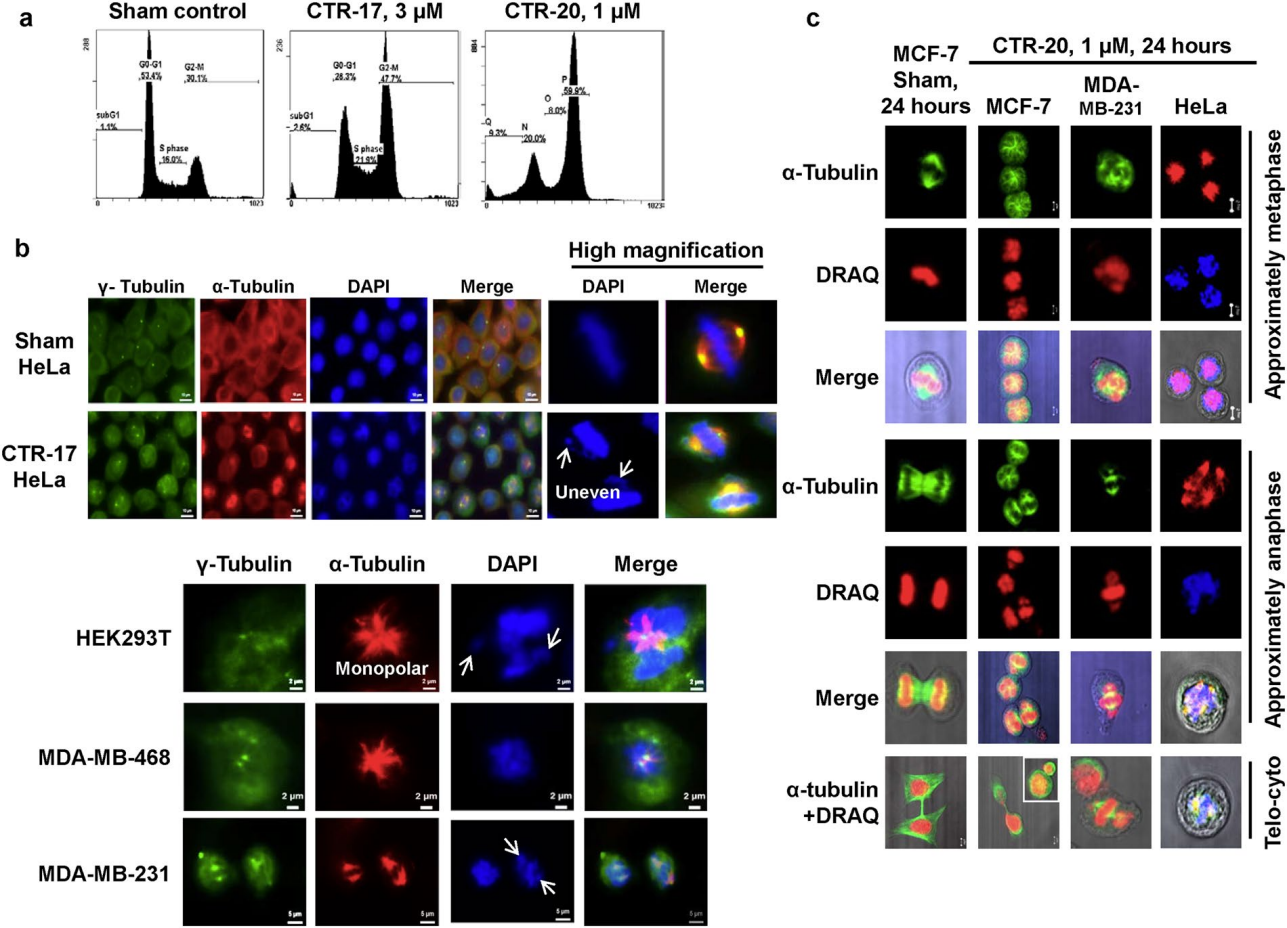
CTRs caused defects in centrosome positioning and chromosome segregation, leading to cell cycle arrest at mitosis. (**a**) MDA-MB-231 cells were sham treated or treated with 3.0 µmol/L CTR-17 or 1.0 µmol/L CTR-20 for 24 hours, and analyzed for their cell cycle profiles by flow cytometry. (**b**) Four different cell lines (HeLa, HEK293T, MDA-MB-468 and MDA-MB-231) were treated with 3.0 µmol/L CTR-17 for 12 hours, fixed in methanol, and immunostained with an antibody specific for α-tubulin (red) or γ-tubulin (green), followed by counterstaining with DAPI (blue). White arrows show the failure of proper alignment or uneven segregation of chromosomes. (**c**) Cells were treated with 1.0 µmol/L CTR-20 for 24 hours, fixed with methanol, and incubated with an antibody specific for α-tubulin (green or red), which was then counterstained with DRAQ5 (red or blue). The internal box shows uneven cell division. “Telo-cyto” denotes cells at the telomere-cytokinesis stage. Statistical data are shown in Supplementary Fig. S8.

DNA profiles obtained by flow cytometry showed that cells treated with CTRs appeared arrested at G2/M at 24 hours post-treatment (Fig. 4a). A more detailed study with synchronized HeLa cells showed that they were actually arrested at the prometaphase-metaphase transition in the presence of CTR-17 or CTR-20 (Fig. 4b,c; Supplementary Figs S8 and S9 – see below). Data from individual cells immunostained with antibodies specific for α-tubulin or γ-tubulin showed that both CTR-17 and CTR-20 caused defects in the chromosome alignment at the center plate of all four different cancer cell lines examined (Fig. 4b,c), which prompted us to measure the distance between two centrosomes in individual cells. As shown in Supplementary Fig. S8b, the distance between two centrosomes in the cells treated with 3.0 μmol/L CTR-17 was 35% shorter, compared to the sham-treated control (i.e., 4.83 ± 0.36 nm *vs* 7.46 ± 0.14 nm). This suggests that CTRs may cause impediment of centrosome migration to the polar region, probably by disrupting microtubule activities.

To gain a better understanding about the cell cycle arrest at mitosis, we carried out flow cytometry and Western blotting with cells synchronized at the G1/S border by double thymidine (DT) treatment and then released into the cell cycle in the absence or presence of CTR-17. As shown in Supplementary Fig. S9a, cells reached G2/M by 6 hours post-G1/S for both sham-treated and 3.0 μmol/L of CTR-17-treated samples, indicating that CTR treatment did not impede cell cycle progression in S phase. Sham-treated control progressed into G1 of the next cell cycle by 9 hours post-G1/S, and cell division process of the entire population was completed by 12 hours post-G1/S. In contrast, cells treated with 3.0 μmol/L of CTR-17 were arrested at least until 16 hours post-G1/S, leading to cell death with sub-G1 DNA content by 48 hours-post G1/S (Supplementary Fig. S9a).

Data from Western blotting showed that the Tyr15 residue was completely dephosphorylated by 12 hours post-G1/S in cells treated with 3.0 μmol/L CTR-17, coinciding with the elevated levels of Cdc25C phosphorylation on Thr48 (Supplementary Fig. S9b). Since Thr161 is highly phosphorylated at the same time points, this data suggests that Cdk1 was already activated by Cdc25 phosphatase (i.e., dephosphorylated Tyr15 on Cdk1) after 9 hours post-G1/S. We noted that the level of securin was still high up to at least 20 hours post-G1/S, indicating that chromosomes were not segregated by that time point. Together, these data are consistent with the notion that cell cycle was arrested at SAC. This conclusion is strengthened by the fact that cyclin B level was still high until 20-hour post-G1/S while no cyclin E and only a low level of cyclin A were detected at 9–20 hours post-G1/S. The high level of phosphorylated histone H3 is also consistent with the high activity of Cdk1/cyclin B kinase.

To further confirm that CTRs caused prolonged SAC, we carried out co-immunoprecipitation to determine APC/C activation. Our data showed that that BubR1was associated with Cdc20, indicating that APC/C was still inactive by 12 hours post-G1/S, resulting in prolonged SAC activation (Supplementary Fig. S10a). Furthermore, BubR1 is accumulated at the kinetochores in the cells treated with 3.0 μmol/L CTR-17 for 12 hours (Supplementary Fig. S10b). Thus, we have concluded that HeLa cells are arrested at SAC in the presence of CTR-17.

### CTRs inhibit microtubule polymerization by binding to β-tubulin at colchicine-binding pocket

**Figure 5:**
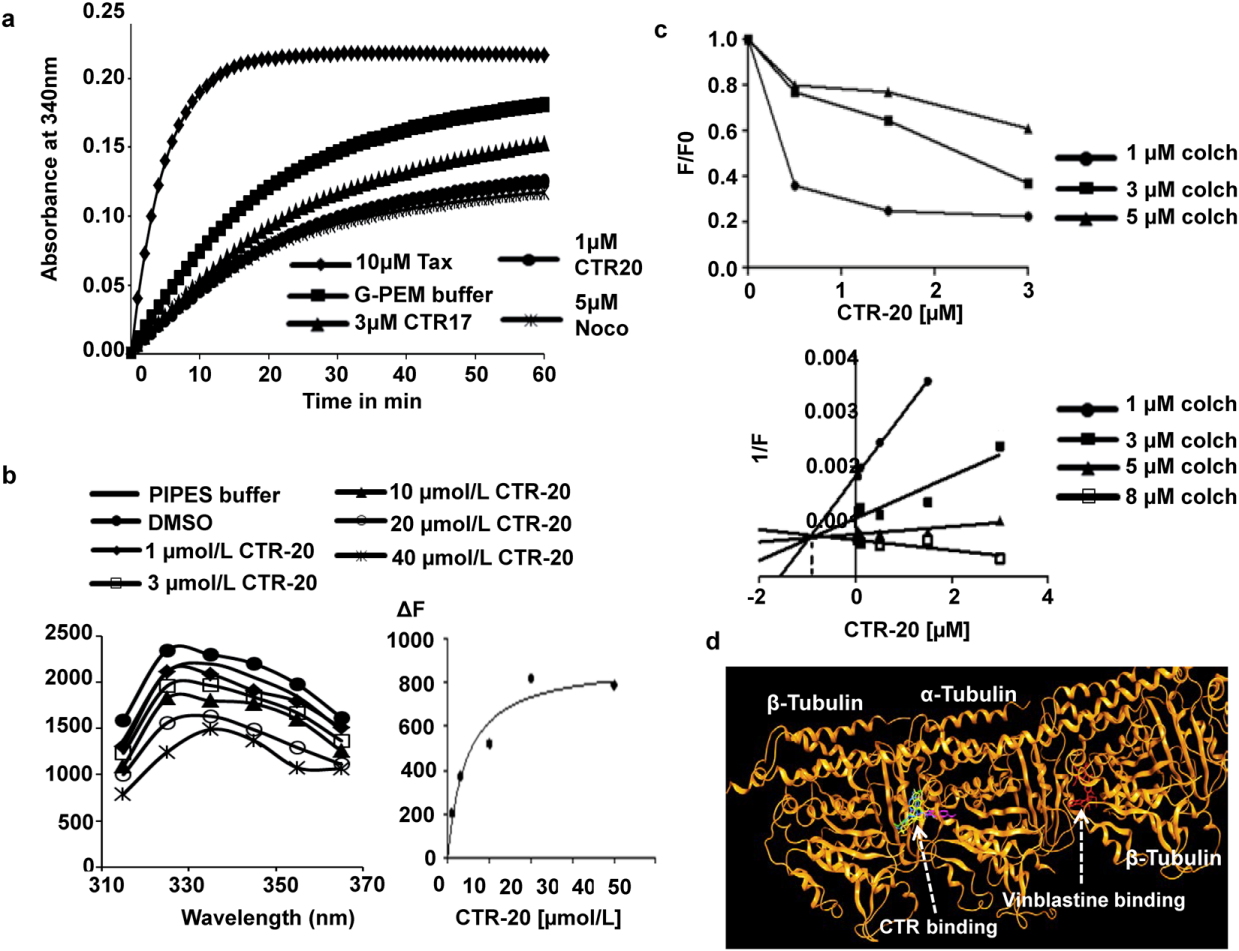
CTRs inhibited microtubule polymerization by binding to tubulin through the colchicine-binding pocket. **(a**) CTRs inhibited microtubule polymerization in a similar manner to that of nocodazole. Purified porcine tubulin and 1.0 mmol/L GTP were added to reaction mixture (37 °C) containing 10 µmol/L paclitaxel (Tax), 3 µmol/L CTR-17, 1 µmol/L CTR-20, or 5 µmol/L nocodazole (Noco). Polymerization of microtubule was monitored every minute for one hour at 340 nm by spectrophotometry. Note that G-PEM buffer contains 400 µg of purified tubulin. (**b**) CTR-20 (and CTR-17; Supplementary Fig. S12) bound tubulin. CTR-20 quenched the intrinsic tryptophan fluorescence of tubulin in a dose-dependent manner. The changes in fluorescence intensity (∆F) were plotted against drug concentrations to determine the dissociation constant. Data are an average of five independent experiments. (**c**) CTR-20 inhibited the binding of colchicine to tubulin. CTR-20 (and CTR-17; Supplementary Fig. S12) depressed the fluorescence of the colchicine-tubulin complex in a dose-dependent manner. The fluorescence intensity of the final tubulin complex was used to determine the inhibitory concentration (Ki) using a modified Dixon plot. The fluorescence intensity was normalized by subtracting any intrinsic fluorescence of CTR-20 at given doses from that of the complex. “F” is the fluorescence of the CTR-colchicine-tubulin complex, and “F0” is the fluorescence of the colchicine-tubulin complex. Data are an average of at least four independent experiments. (**d**) The tubulin-binding sites of CTR-17 (green) and CTR-20 (magenta) closely overlap with those of colchicine (blue) and podophyllotoxin (yellow). For detail, see Supplementary Figs S12 and S13. Vinblastine binding site is shown as a reference.

Since data shown in Fig. 4 and Supplementary Fig. S8 were consistent with the notion that CTRs may impede microtubule functions, we examined the microtubule polymerization activity in the absence or presence of CTRs. Data from an *in vitro* microtubule assembly assay showed that the light scattering pattern in the presence of CTR-17 or CTR-20 was similar to that of nocodazole treated samples but not that of paclitaxel, indicating that both CTRs may inhibit microtubule polymerization (Fig. 5a). Data obtained from Western blotting with soluble and polymer fractions from three different cell lines (HeLa, MDA-MB-231 and MDA-MB-468) are also consistent with data from the light scattering assay (Supplementary Fig. S11).

Intrinsic tryptophan fluorescence of tubulin is widely used as a probe to determine the binding affinity of drugs to tubulin heterodimers^29–32^. We found a concentration-dependent quenching of the tryptophan fluorescence when purified tubulin was incubated with CTR-17 (Supplementary Fig. S12a) or CTR-20 (Fig. 5b). Changes in the fluorescence intensity were calculated relative to that of sample containing tubulin with PIPES buffer only, and the binding constants (Kd) were determined by fitting-in the fluorescence changes^29^ in a binding isotherm for CTR-17 or CTR-20. The Kd values were 4.58 ± 0.95 µmol/L and 5.09 ± 0.49 µmol/L for CTR-17 and CTR-20, respectively.

Since certain chalcone derivatives may bind to β-tubulin through the colchicine binding site^20–24^, we determined if it is the case for CTRs. For this study, we took advantage of the fact that: (i) the fluorescence intensity of colchicine increases as they bind to tubulins^33, 34^; and (ii) the binding of CTR to the same binding pocket of colchicine can displace colchicine molecules from the colchicine-tubulin complexes, resulting in the reduction of fluorescence intensity. As expected, the addition of either CTR-17 or CTR-20 to the reaction mixture containing colchicine-tubulin complexes could reduce fluorescence in a dose-dependent manner, indicating that both CTRs bind to the tubulin at the colchicine-binding pocket (Fig. 5c; Supplementary Fig. S12b).

When a modified Dixon plot was generated to determine the Ki values, the trend lines drawn for each colchicine concentration intersects above the X-axis at a single point, indicating that the mode was competitive inhibition^35^. This data again suggests that CTRs can compete with colchicine for tubulin binding. However, the Ki values indicate that CTR-20 binds to the colchicine-binding pocket approximately five times more strongly than CTR-17 does (Fig. 5c; Supplementary Fig. S12b, lower panels).

To gain a further understanding about the tubulin binding by the CTR compounds, we carried out an *in silico* docking study using MOE (see Supplementary Information). The resultant data showed that the binding sites for both CTR-17 and CTR-20 partially overlap with that of colchicine on β-tubulin at the interface with α-tubulin (Fig. 5d). Both CTR-17 and CTR-20 are likely stabilized by covalent binding and non-covalent interactions (Supplementary Figs S12c and S13). CTR-20 forms two H-bonds and one arene-cation interaction with the tubulin moiety, while CTR-17 forms only a single H-bond, supporting the biochemical evidence that CTR-20 binds to the colchicine-binding site more tightly than CTR-17. In spite of striking differences in the structures, colchicine, podophyllotoxin and the two CTR compounds occupy the same binding pocket on the tubulin, as shown in the overlapping docking images (Supplementary Figs S12c and S13).

### CTR-20 is not a substrate of MDR1 or MRP1, but inhibits MRP1 activity

Intriguingly, despite both colchicine and CTRs target the same binding pocket on the β-tubulin at the interface with α-tubulin (Fig. 5; Supplementary Figs S12 and S13), CTRs effectively killed multidrug-resistant cells while colchicine did not (Fig. 2). One plausible explanation may be that, unlike colchicine, CTRs may be substrates of neither MDR1 nor MRP1. Another possibility is that CTR may impede the efflux pumps. In fact, there was a report that chalcone derivatives could inhibit MDR and MRP functions without being substrates^36^.

**Figure 6:**
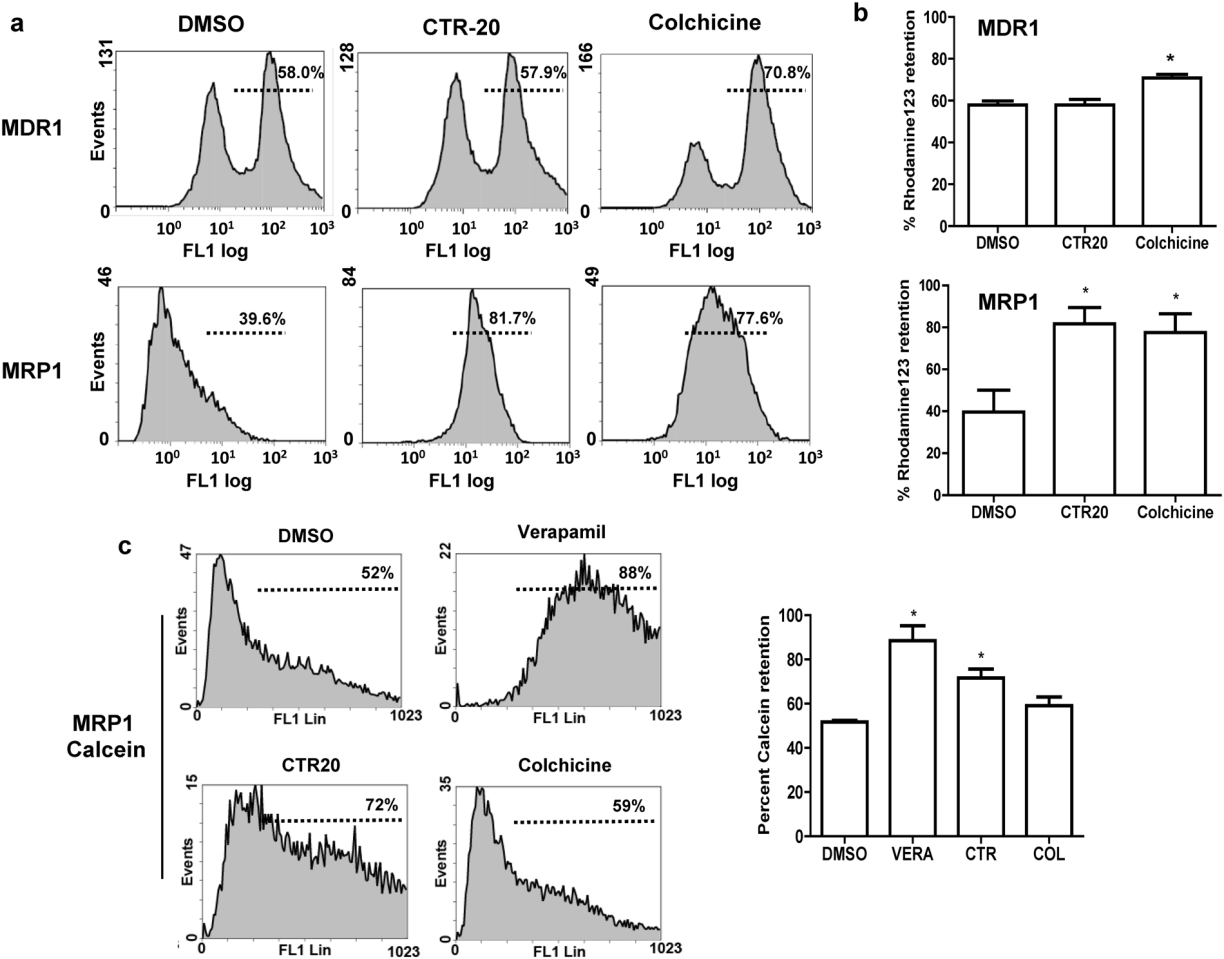
CTR-20 overcomes multidrug-resistant cells by two different molecular mechanisms. (**a**,**b**) Data from Rh123 dye retention indicated that CTR-20 did not inhibit MDR1-dependent efflux, but did prevent MRP1-dependent efflux. The efflux assays were carried out with the K-B-C2 (MDR1) or HEK293-MRP1 (MRP1) multidrug resistant cells using a Rh123 drug efflux analysis system as described in Methods. Each histogram shown in panel b is from three independent experiments, and the average percent of retention is shown in each diagram in panel a. DMSO denotes a vehicle-only control. (**c**) CTR-20 inhibits MRP1-dependent efflux of calcein-AM. HEK293-MRP1 cells were incubated with calcein-AM in the presence of CTR-20, colchicine, verapamil (positive control) or a vehicle control. The data is also shown in a histogram. Data presented are mean ± S.E.M value of three independent experiments. 1-way ANOVA was carried out with post-hoc Dunnett’s test performed comparing each drug against vehicle control (*p < 0.05).

To determine whether CTR-20 is a substrate of MDR1 or MRP1, we used an indirect Rh123 efflux assay where the retained levels of Rh123 would be higher by competition for the efflux pump if CTR-20 is a substrate of the efflux pump. Data from an assay carried out with the MDR1-overexpressing KB-C-2 cells showed that CTR-20 did not prevent the efflux of Rh123 above the control level (58.0% *vs* 57.9% in Fig. 6a,b), suggesting that CTR-20 is not a substrate of MDR1. In contrast, the treatment of colchicine resulted in the higher level of Rh123 retention in the cells (58.0% *vs* 70.8% in Fig. 6a,b), which is consistent with the previous report that colchicine is an MDR1 substrate^37^. Performing a similar assay in HEK293-MRP1 cells, we found that the treatment of CTR-20 or colchicine resulted in the much higher levels of Rh123 in the cells, compared to the DMSO control (39.6% *vs* 81.7% or 39.6% *vs* 77.6% in Fig. 6a,b). At a glance, this data appeared to indicate that both CTR-20 and colchicine are substrates of MRP1. Indeed, colchicine was previously found to be an MRP1 substrate^38^. However, CTR-20 is not likely a substrate of MRP1 since, unlike colchicine, it was effective against HEK293-MRP1 (Fig. 2b). Therefore, we thought that the high levels of Rh123 retention in the MRP1-overexpressing cells in the presence of CTR-20 or colchicine might be by two different mechanisms: colchicine as being an MRP1 substrate and CTR-20 as being an inhibitor.

To examine this possibility, we carried out a calcein-AM retention assay to directly measure the inhibitory effects of CTR-20 and colchicine on MRP1. We found that the level of calcein-AM retained in the cells treated with CTR-20 was 72%, which was an intermediate level of the DMSO control (52%) and verapamil (a known MRP1 inhibitor) (88%). In contrast, the calcein-AM level of cells treated with colchicine was similar to that of the negative control (52% *vs* 59%) (Fig. 6c). Taken together, data shown in Fig. 6 is consistent with the notion that CTR-20 is neither a substrate of MDR1 nor MRP1, while colchicine is substrate for both. Furthermore, CTR-20 is an inhibitor of the MRP1 efflux pump, explaining that CTRs, but not colchicine, are effective against both MDR1- and MRP1-overexpressing multidrug resistant cells, even if they bind to the same colchicine-binding pocket.

### The effects of CTRs are reversible

As presented above, our data showed that cells arrested at the SAC stage^39^ in the presence of CTR-17 or CTR-20 (Fig. 4; Supplementary Figs S8, S9 and S10). This prolonged cell cycle arrest in turn activated apoptosis (Supplementary Figs S6 and S9a). To determine if the CTR-mediated cell cycle arrest is reversible, we released cells (HeLa) into drug-free complete medium after they were treated with 3.0 μmol/L CTR-17 or 1.0 μmol/L CTR-20 for 12 hours. Within 3 hours of release, a significant portion of cell population progressed into G1 of the next cell cycle (Supplementary Fig. S14). By 9 hours of the release, most cells moved out of the M phase arrest. Thus, we have concluded that the effects of CTR-17 and CTR-20 are reversible.

### CTR-17 and CTR-20 showed effective antitumor activities in mice without showing notable side effects to animals

**Figure 7:**
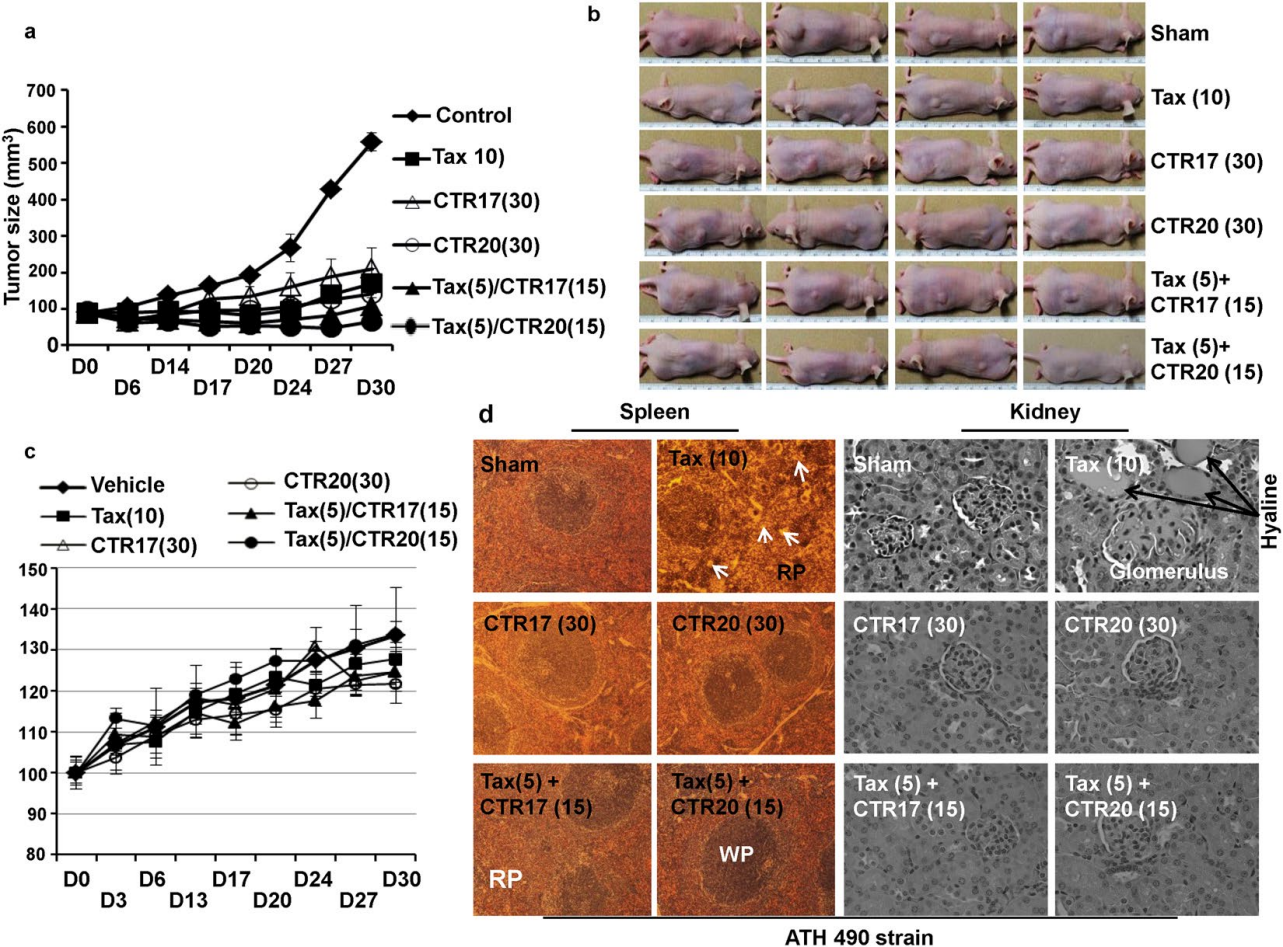
CTRs are effective anti-tumor agents. (**a**) Changes in tumor size (mm^3^) in response to CTR-17, CTR-20 or paclitaxel (Tax) are shown. Tumors were induced in ATH490 athymic mice with MDA-MB-231 cells. Treatments started when tumor volume reached ~90 mm^3^. “D” denotes day(s) post-treatment. Numbers in brackets are mg/kg body weight. The raw data of tumor volumes are shown in Supplementary Table S3. (**b**) The representative pictures of ATH490 mice with tumors that were vehicle (sham)-treated or treated with drugs as described in panel a. (**c**) Body weights of mice (CD1 strain) were not affected by treatments with CTR-17 or CTR-20 at doses used. Normalization of the body weights was based on the body weight on day 0 as 100%. (**d**) Spleens and kidney stained with H & E are shown. White arrows in spleen indicate the presence of macrophages in the red pulp (RP) in mice treated with paclitaxel (10 mg/kg). Hyaline is accumulated in the kidney of mice treated with Tax (10 mg/kg). Images were taken with a Zeiss EPI-fluorescent microscope at 20× , 10× and 40× objectives for liver, spleen, and kidney, respectively.

We determined the antitumor activity of CTR-17 and CTR-20 using ATH490 athymic mice engrafted with MDA-MB-231 triple-negative metastatic breast cancer cells. The treatment of tumor-bearing mice with CTR-17 or CTR-20 (30 mg/kg body weight, i.p.) twice per week for 30 days effectively suppressed tumor growth (Fig. 7a,b; Supplementary Tables S2 and S3). When used in combination with a ½-dose (i.e., 5 mg/kg) of paclitaxel, a ½-dose (15 mg/kg) of CTR-17 or CTR-20 showed substantially improved antitumor activity (Fig. 7a; Supplementary Table S3).

We then determined the changes in body and organ weights in response to the drug treatments. Mice treated with CTR-17 (30 mg/kg), CTR-20 (30 mg/kg), paclitaxel (10 mg/kg), or the combination of CTR and paclitaxel in half doses did not cause any notable side effects on either CD-1 or ATH490 strains (Fig. 7c; Supplementary Fig. S15). To examine potential liver toxicity by CTRs, we made visual observations with H&E stained liver tissues. We did not find any increase of mitotic index in the livers of either CD-1 or ATH490 treated with CTR-17, CTR-20, or CTR-paclitaxel combinations (in half doses), indicating that these treatments did not cause any notable liver injuries (Fig. 7d; Supplementary Fig. S16). We also measured the levels of serum ALT and AST. The levels of ALT and AST of the vehicle-treated and those treated with CTR-17 (30 mg/kg), CTR-20 (30 mg/kg), or combinations of paclitaxel (5 mg/kg) and CTR (15 mg/kg) were in the normal range for CD1 mice: 38.76 ± 0.17 (vehicle treated), 30.25 ± 0.73 (10 mg/kg paclitaxel), 37.02 ± 1.46 (30 mg/kg CTR-17), 30.43 ± 0.49 (30 mg/kg CTR-20), 22.33 ± 0.23 (5 mg/kg paclitaxel plus 15 mg/kg CTR-17), and 36.74 ± 0.70 (5 mg/kg paclitaxel plus 15 mg/kg CTR-20). The AST and ALT levels were generally higher for ATH490 strain; however, the levels were in the same range for controls and drug-treated mice (Supplementary Fig. S16b). Thus, liver toxicity measured with the levels of AST and ALT also indicated that CTR-17 and CTR-20 alone or in combination with paclitaxel in half doses did not cause any notable ill-effects to animals.

CTR compounds did not cause any detectable spleen toxicity when used alone or in combinations with paclitaxel in half doses (Fig. 7d; Supplementary Fig. S16c, Spleen). However, animals treated with 10 mg/kg of paclitaxel showed an increase in either white pulp (CD1 mice; Supplementary Fig. S16c) or red pulp (ATH490 strain; Fig. 7d) in approximately 20% of the cases, indicating that paclitaxel at this dose may cause a minor inflammatory response to spleen. We found that neither CTR-17 nor CTR-20 caused any ill-effects on kidney morphology (Fig. 7d; Supplementary Fig. S16c, Kidney). In contrast, one in four mice treated with 10 mg/kg paclitaxel showed the presence of hyaline, suggesting that high levels of paclitaxel may cause minor pathological effects on kidney (Fig. 7d). At the cellular level, no notable morphologic changes were observed in lungs of animals treated with CTR-17, CTR-20, paclitaxel, or the CTR-paclitaxel combinations in half doses (data not shown).

## Discussion

Based on the postulation that certain chalcone-derivatives can effectively overcome the multidrug-resistant problem with minimal side effects^20, 26^, we synthesized and examined a small chemical library comprising 24 novel quinolone chalcones. We found that these chemicals were generally quite potent in killing cancer cells (which will be reported elsewhere along with the detailed procedure of chemical synthesis). We then chose to study CTR-17 and CTR-20 in more detail as they effectively and preferentially killed a wide range of cancer over non-cancer cells. Our study included two non-cancer cell lines (MCF 10A and 184B5) and 65 cancer cell lines originated from 12 different tissues: leukemia (6 lines), lung (10 lines), brain (2 lines), colon (7 lines), CNS (6 lines), melanoma (9 lines), ovary (7 lines), kidney (8 lines), prostate (2 lines), breast (6 lines), urothelial (1 line) and cervix (1 line). In addition, several multidrug-resistant cell lines were also used to examine drug efficacy and synergistic effects. Our data and the study carried out in collaboration with the US National Cancer Institute (Supplementary Figs S1–S5) showed that CTR-20 is very effective against all of the cancer cell lines examined.

We also found that, in stark contrast to three well-known tubulin-targeting drugs (paclitaxel, colchicine and vinblastine), both CTR-17 and CTR-20 effectively killed MDR1- and MRP1-overexpressing multidrug-resistant cells originated from three different tissues: cervix (KB-C-2), kidney (HEK293-MRP1) and breast (MDA-MB-231TaxR) (Fig. 2). In particular, both CTR-17 and CTR-20 were highly effective in killing MDA-MB-231TaxR, a cell line 114 times more resistant to paclitaxel. Furthermore, the combination of paclitaxel and CTR-17 or CTR-20 showed synergistic effects against two different multidrug-resistant cancer cell lines examined (KB-C-2 and MDA-MB-231TaxR) (Fig. 2). Considering the fact that paclitaxel, one of the most widely used anticancer drugs, is associated with high toxicity and multidrug-resistance issue, the CTR compounds, alone or in combination with paclitaxel, can be an excellent solution to overcome the shortcoming of paclitaxel. Moreover, CTR-20 showed extremely strong synergistic effect against the MDA-MB-231 triple-negative metastatic breast cancer cells when combined with a low dose of ABT-737 (Fig. 3). ABT-737, an inhibitor of Bcl-2 family proteins, is effective against myeloid leukemia^40^. Unfortunately, it provokes transient thrombocytopenia through the inhibition of Bcl-X_L_, an essential survival factor for platelet cells^28, 41^. This finding led to the development of the Bcl-2 specific inhibitor ABT-199, which is effective against leukemia without provoking thrombocytopenia^42–44^. However, ABT-199 is not effective against solid tumors^45, 46^, which is consistent with the finding that high levels of Bcl-X_L_ are found in a wider range of solid tumors^45, 47–51^. Thus, ABT-737 has better potential in controlling solid tumors, notably against small cell lung cancer^45^. In general, though, ABT-737 is not very effective against solid tumors as a single agent^52^. Since the combination of ABT-737 and CTR-20 is extremely synergistic against MDA-MB-231 (CI = 0.07), and since ABT-737 at the dose used in our experiment did not activate Bcl-X_L_ (Fig. 3a,c), the combination of these two compounds can be highly promising for the treatment of solid tumors.

Both CTR-17 and CTR-20 effectively killed the MDR1-overexpressing KB-C-2 and the MRP1-overexpressing HEK293-MRP1 cells, while colchicine is not effective (Fig. 2). This finding is surprising since the CTR compounds share their binding sites with colchicine (Fig. 5d; Supplementary Figs S12c and S13). As the binding patterns of colchicine and the CTRs to β-tubulin are only slightly different, this difference alone cannot likely explain why their efficacies against multidrug-resistant cancers are dramatically different. One possible explanation may be that colchicine is an effective substrate of MDR1 and MRP1 while CTR compounds are not. Indeed, our data is consistent with the notion that, unlike colchicine, CTR-20 is a substrate of neither MDR1 nor MRP1 (Fig. 6). Furthermore, CTR-20 actually impedes the MRP1 efflux activity (Fig. 6c). Thus, CTRs can be extremely effective drugs in controlling multidrug-resistant tumors as they can effectively overcome drug resistance.

It was shown previously that colchicine is very toxic to human^4^. Unlike colchicine, CTR compounds did not show any notable side effects in animals (Fig. 7c; Supplementary Figs S15 and S16). This may be, at least in part, relevant to the fact that CTRs are reversible (Supplementary Fig. S14). Thus, the CTR compounds can be anticancer drugs superior to colchicine and, possibly, other known tubulin inhibitors.

We note that the number of H-bonds between the three compounds (CTR-17, CTR-20 and colchicine) and amino acid residues of β- tubulin may be relevant to differences in efficacy, reversibility and toxicity. In this aspect, CTR-20, which can form two H-bonds, may be advantageous over CTR-17 (one H-bond) and colchicine (three H-bonds), as CTR-20 is highly effective against many different cancers (Fig. 1; Supplementary Figs S1–S5). Furthermore, CTR-20 showed strong synergy with paclitaxel and ABT-737. The antitumor potency of CTR-20 (30 mg/kg by i.p.) is comparable to that of paclitaxel (10 mg/kg by i.v.), without causing a notable side effect. Moreover, the combination of a ½ dose of CTR-20 and a ½ dose of paclitaxel was more effective than full dose of either CTR-20 or paclitaxel alone, without causing any notable side effects. Therefore, this data raises the possibility that the combination of CTR and paclitaxel (and possibly ABT-737) can achieve better therapeutic results even against multidrug-resistant tumor, with minimum side effects.

## Methods

### Cell lines and cell culture

All cancer and non-cancer cell lines were purchased from American Tissue Culture Collection (ATCC) (Manassas, VA) and cultured according to supplier’s instructions. The list of cell lines used, authentication and culture conditions are described in Supplementary Information.

### Microtubule assays

The effect of CTR compounds on microtubule polymerization was carried out with a tubulin polymerization kit (BK004P, Cytoskeleton). The assay was adapted from the method described previously^53^. Absorbance was measured with an automated plate reader (Synergy H4 Hybrid Multi-Mode Microplate Reader) for one hour at one-minute intervals. The experiment was performed in triplicates and repeated at least two times.

A two-step extraction procedure was used to differentially extract tubulin heterodimers and microtubules from sham-treated cells or those treated with compounds as described previously^54^. To determine the binding ability of CTR compounds to tubulin, the changes in the intrinsic tryptophan fluorescence of purified porcine tubulin (T240, Cytoskeleton) was measured as described previously^29^. GraphPad Prism software was used to determine the dissociation constant of CTRs bound to tubulin, and the following formula was used:

$$\triangle F=\frac{\triangle F{\max }\times C}{{Kd}+C}$$

Where, ΔF is the changes in the fluorescence intensity of the tubulin when bound by CTR; ΔFmax is the maximum change in the fluorescence intensity; C is compound concentration; and Kd is the dissociation constant of the compound. The experiment was repeated five times.

To determine the mode of binding, a competitive binding assay was carried out as described previously^29^. Briefly, tubulin (0.4 μmol/L) was incubated with different concentrations of CTR for 1 hour at 37 °C. Subsequently, colchicine was added to the reaction mixture containing CTR-tubulin complexes. To determine inhibition constant (Ki), colchicine was added to a final concentration of 3.0 μmol/L, 5.0 μmol/L or 8.0 μmol/L to different concentration of the CTR-17-tubulin complex, and 1.0 μmol/L, 3.0 μmol/L, 5.0 μmol/L or 8.0 μmol/L to different concentration of the CTR-20-tubulin complex. Fluorescence was monitored using an automated plate reader (Synergy H4 Hybrid Multi-Mode Micro plate Reader) with an excitation wavelength at 360 nm and emission at 430 nm. A modified Dixon plot was used to analyze the competitive inhibition of colchicine binding to tubulin, and to determine the Ki of CTR-17 and CTR-20. The experiment was repeated four times.

### Dye efflux Assays

Rhodamine123 (Rh123) drug efflux was carried out using a Multidrug-Resistance Direct Dye Efflux Activity Kit (Millipore, Darmstadt, Germany), with some modifications. 2.5 × 10^5^ cells were collected and incubated at 4 °C for 1 hour in cold efflux buffer supplemented with 10 µg/mL of Rh123 and 22 µmol/L of drug (CTR-20, colchicine) or a vehicle control. Cell pellets were washed with cold efflux media containing sham control or 22 µmol/L of drug, and resuspended in pre-warmed efflux media containing 22 µmol/L of drug (or vehicle) and incubated for 1 hour in 37 °C water bath. Cells were then placed on ice and fluorescence levels were quantified by flow cytometry using a Beckman Coulter FC500 flow cytometer, 488 nm excitation, FL1 (525 nm/40) emission. Calcein-AM (Santa Cruz, Dallas) efflux was carried out on 1 × 10^7^ cells, which were co-treated with sham control or 22 µmol/L of CTR-20 (or colchicine/verapamil) and 0.5 µmol/L calcein-AM for 15 min at 37 °C on 10 cm plates, followed by washing/pelleting with ice-cold media. Reading was carried out with a Beckman Coulter FC500 flow cytometer.

### Animal work

Five-week old female CD-1 (strain code 022) and ATH490 (strain code 490) athymic nude mice were purchased from Charles River (Quebec, Canada). All animal experiments, including animal handling, care, treatments and endpoint determination were reviewed and approved by the Animal Care Committee (ACC) at Laurentian University (Sudbury, Ontario, Canada). We hereby confirm that all experiments were performed in accordance with relevance guidelines and regulation.

For paclitaxel treatments, 40 mg/ml stock solution of paclitaxel (Sigma, MO) was prepared in DMSO. Just before administration to mice, the stock solution was diluted 10-fold in buffer containing 10% DMSO, 12.5% cremophor, 12.5% ethanol, and 65% saline-based diluent (0.9% sodium chloride, 5% polyethylene glycol, and 0.5% tween-80), which is defined as vehicle^55, 56^. Alanine transaminase (ALT, SUP6001-c) and Aspartate transaminase (AST, SUP6002-c) color endpoint assay kits were purchased from ID Labs (London, Ontario, Canada).

Athymic mice engrafted with tumor cells were used to determine antitumor activity. Briefly, exponentially growing (MDA-MB-231 metastatic breast) cancer cells (10 × 10^6^ cells in 0.2 ml ice-cold PBS) were injected into the flank of mice. When tumor size reached 5–6 mm in diameter (n = 4–5 per group), mice were randomly assigned into several groups and vehicle-treated or treated with drug(s). The detailed treatment schedule is shown in Supplementary Table 2 and as described previously^56^. Animals were monitored for food and water consumption every day, and their body weights and tumor volumes were measured twice per week. Tumor volumes were measured with a digital caliper and were determined by the following formula: ½ length × width^2^. Blood samples were collected via cardiac puncture and processed for ALT and AST measurements. The animals were then immediately euthanized by carbon dioxide. Tumors and vital organs (spleen, kidney, liver and lung) were harvested and fixed in 10% buffered formalin at 4 °C overnight before being processed for a paraffin embedding. The paraffin-embedded blocks were then sectioned to 4–5 µm thick. Each section was stained with haematoxylin and eosin (H&E).

### Statistical Analyses

All values are expressed as mean ± S.E.M of at least three independent experiments. Analyses were performed using GraphPad Prism software (v5). To determine if the distance between the centrosomes of sham-treated or CTR-treated cells are significantly different, an unpaired t-test was performed and a p-value of <0.0001 was considered to be statistically significant. All values are mean ± S.E.M of at least three independent experiments, unless stated otherwise. Comparison between groups was made by P value using one-way ANOVA: p < 0.05 was considered to be statistically significant.

### Data availability

Data presented in this paper are available to the scientific community.

## Electronic supplementary material

Supplementary Information
