# Supplementary material for: Novel quinolone chalcones targeting colchicine-binding pocket kill multidrug-resistant cancer cells by inhibiting tubulin activity and MRP1 function
Source: Sci Rep. 2017 Aug 31;7:10298. doi: 10.1038/s41598-017-10972-0 (PMC5578999; doi:10.1038/s41598-017-10972-0)
Supplement: Supplementary file 1 — Supplementary Information [file 41598_2017_10972_MOESM1_ESM.pdf]

Supplementary Information

Novel quinolone chalcones targeting colchicine-binding pocket kill multidrug-resistant cancer cells by inhibiting tubulin activity and MRP1 function

I. Kalhari Lindamulage[1, 2†], Hai-Yen Vu[1†], Chandrabose Karthikeyan[4], James Knockleby[1], Yi-Fang Lee[1], Piyush Trivedi[4], and Hoyun Lee[1,2,3*]

[1] Health Sciences North Research Institute, 41 Ramsey Lake Road, Sudbury, Ontario P3E 5J1, Canada
[2] Biomolecular Sciences, Laurentian University, 935 Ramsey Lake Road, Sudbury, Ontario P3E 2C6, Canada
[3] Departments of Medicine, the Faculty of Medicine, the University of Ottawa, Ottawa, Ontario K1H 5M8, Canada
[4] School of Pharmaceutical Sciences, Rajiv Gandhi Technical University, Airport Bypass Rd, Gandhi Nagar, Bhopal, M.P, India

* Corresponding. hlee@hsnri.ca
† These two authors contributed equally to this work.

Supplementary Methods

Cells and cell culture

The cell lines used for this study are as follows: MDA-MB-231 (undifferentiated, triple-negative breast adenocarcinoma), MDA-MB-468 (intermediately differentiated, triple-negative breast adenocarcinoma), MCF-7 (differentiated, ER+ breast adenocarcinoma), K562 (chronic myelogenous leukemia [CML]), RPMI-8226 (multiple myeloma), U87MG (glioblastoma with hypo-chromosome number of 43-45), T98G (temozolomide-resistant glioblastoma with hyper chromosome number of 128-132), HEK293T (human embryonic kidney cells with constitutively expressing SV40 T-ag), UC3 (human urothelial carcinoma), A549 (human lung carcinoma), NCI-H1975 (non-small cell lung adenocarcinoma) and HeLa (cervical adenocarcinoma). In addition, the entire collection of the US NCI-60 cancer panel was used to examine the efficacy of CTR-20. The 184B5 and MCF10A are non-cancer immortalized breast epithelial call lines. Two isogenic MCF10A cell lines were also used: MCF10AT1 is a premalignant cell line generated by transforming MCF10A with c-Ha-Ras (Basolo et al., 1991); and MCF10CA1a cell line was isolated by selecting malignant cells after MCF10AT1 was engrafted into mice (Marella et al., 2009; Santner et al., 2001). The MCF10AT1 and MCF10CA1a were kind gifts of Dr. Valerie Weaver at the Center for Bioengineering and Tissue Regeneration, UCSF, CA. The MCF10A

and MCF10AT1 cells were cultured in Dulbecco's Modified Eagle's Medium/Ham's Nutrient Mixture F-12 (DME/F12) supplemented with 10% (volume/volume) FBS, 100 µg/ml streptomycin and 100 units/ml penicillin, antimycotic solution, 0.5 µg/ml hydrocortisone, 10 ng/ml human epidermal growth factor (hEGF) and 5 µg/ml insulin, whereas MCF10CA1a cell line was cultured in DMEM supplemented with 10% FBS (volume/volume). KB-3-1, parental human epidermoid carcinoma cell line and KB-C2, an isogenic MDR1-overexpressing drug-resistant cell line, were kindly provided by Dr. Amit K Tiwari, the University of Toledo, OH. Parental HEK293 and MRP1-overexpressing HEK293-MRP1 isogenic cells were kind gifts of Dr. Susan Cole (Queen's University, Kingston, Ontario, Canada). The paclitaxel-resistant MDA-MB-231TaxR cell line was generated in house by culturing MDA-MB-231 cells (purchased from ATCC and authenticated) in the gradually increasing concentrations of paclitaxel until the concentration reached to 100 nM. Cell line authentication was carried out by Genetica DNA Laboratories (Burlington, NC) using a short tandem repeat (STR) profiling method (March 2015; July 2015; September 2016).

Cell viability, combinational index and cell synchronization

Cell viability and proliferation were examined using a sulforhodamine B (SRB) assay as described previously (Hu et al., 2008). IC_{50} values were calculated from sigmoidal dose-response curves generated by at least two independent experiments, with each quadruplicate samples, using GraphPad Prism v.5.04 software (GraphPad Software, Inc, La Jolla, CA). For combinational experiments on the KB-C-2 cells, CTR compounds and paclitaxel or ABT-737/ABT-199 were used at or below IC_{50}. The combinational index (CI) was calculated as previously described (Chou, 2006). If the CI values are less than, equal to, or more than 1.0, each indicates a synergistic, additive, or antagonistic effect, respectively. Synchronisation at the G1/S border was achieved with a double thymidine (DT) block approach as described previously (Romero and Lee, 2008), and the cells were released into complete medium in the absence (sham) or presence of compound(s).

Cell cycle analysis

Cell cycle progression in the absence (sham) or presence of compound(s) was determined with a Beckman Coulter Epics Elite FC 500 Flow Cytometer as described previously (Romero

and Lee, 2008). The reversibility of drug effects was carried out as follow: HeLa cells treated with CTR-17 or CTR-20 for 12 hours were washed twice with 1× PBS and then released into pre-warmed complete medium. Subsequently, cells were harvested at scheduled time points to examine their DNA profiles/cell cycle positions by flow cytometry.

Immunofluorescence staining was carried out as described previously (Santi and Lee, 2011). Photography was carried out with a Carl Zeiss 510 Meta laser scanning microscope or an Axioscope, and images were captured with LSM image examiner (Carl Zeiss, North York, ON, Canada). For immunostaining, a background control (without secondary antibody) and bleed-through controls (cells labeled with each fluorophore separately) were used to avoid inaccurate interpretation of data due to artifacts. A minimum of 10 fields per coverslip and 200 cells for quantitative cell analysis were captured. Each experiment was repeated at least twice. Western blotting was carried out as described previously (Santi and Lee, 2010).

Molecular Docking

Molecular Operating Environment (MOE) (Chemical Computing Group Inc, Montreal, Quebec, Canada) was used to predict the mode of interactions between compounds and the β-tubulin subunit as described previously (Pundir et al., 2015). The crystal structure of the tubulin-colchicine complex (PDB Code: 1SA0) was used as the target structure and was subjected to energy minimization and protonation. The induce-fit protocol for docking was adopted from that posted in the MOE website (http://www.chemcomp.com/ MOE-Structure_Based_Design.htm). The best docking pose was determined based on the minimum free energy for binding. The contributions of hydrogen (H)-bonds, hydrophobic, ionic and Van der Waals interactions were taken into consideration when calculating binding free energy.

References

Basolo, F., Elliott, J., Tait, L., Chen, X.Q., Maloney, T., Russo, I.H., Pauley, R., Momiki, S., Caamano, J., Klein-Szanto, A.J., *et al.* (1991). Transformation of human breast epithelial cells by c-Ha-ras oncogene. Mol Carcinog *4*, 25-35.

Chou, T.C. (2006). Theoretical basis, experimental design, and computerized simulation of synergism and antagonism in drug combination studies. Pharmacol Rev *58*, 621-681.

Hu, C., Solomon, V.R., Ulibarri, G., and Lee, H. (2008). The efficacy and selectivity of tumor cell killing by Akt inhibitors are substantially increased by chloroquine. Bioorg Med Chem *16*, 7888-7893.

Marella, N.V., Malyavantham, K.S., Wang, J., Matsui, S., Liang, P., and Berezney, R. (2009). Cytogenetic and cDNA microarray expression analysis of MCF10 human breast cancer progression cell lines. Cancer Res *69*, 5946-5953.

Pundir, S., Vu, H.Y., Solomon, V.R., McClure, R., and Lee, H. (2015). VR23: A Quinoline-Sulfonyl Hybrid Proteasome Inhibitor That Selectively Kills Cancer via Cyclin E-Mediated Centrosome Amplification. Cancer Res *75*, 4164-4175.

Romero, J., and Lee, H. (2008). Asymmetric bidirectional replication at the human DBF4 origin. Nat Struct Mol Biol *15*, 722-729.

Santi, S.A., and Lee, H. (2010). The Akt isoforms are present at distinct subcellular locations. Am J Physiol Cell Physiol *298*, C580-591.

Santi, S.A., and Lee, H. (2011). Ablation of Akt2 induces autophagy through cell cycle arrest, the downregulation of p70S6K, and the deregulation of mitochondria in MDA-MB-231 cells. PLoS One *6*, e14614.

Santner, S.J., Dawson, P.J., Tait, L., Soule, H.D., Eliason, J., Mohamed, A.N., Wolman, S.R., Heppner, G.H., and Miller, F.R. (2001). Malignant MCF10CA1 cell lines derived from premalignant human breast epithelial MCF10AT cells. Breast Cancer Res Treat *65*, 101-110.

Supplementary Table S1: CTR-17 and CTR-20 effectively kill multidrug-resistant cells (KB-C-2 & H69AR). Numbers are IC_{50} in nM or μM

	KB-C-1	KB-C-2	Resistance (fold)	SW-1271	H69AR	Resistance (fold)
Colchicine (nM)	5.36±0.54	83.45±7.22	15.57	4.84±0.80	22.97±3.63	4.74
Paclitaxel (nM)	2.01±0.17	23.08±0.21	11.48	4.51±0.71	10.99±2.60	2.44
Vinblastine (nM)	0.61±0.09	9.27±3.22	15.20	1.75±0.21	10.20±1.97	5.82
CTR-17 (μM)	0.38±0.07	0.65±0.16	1.71	1.14±0.04	0.52±0.10	0.45
CTR-20 (μM)	0.10±0.02	0.25±0.03	2.50	1.95±0.01	0.13±0.01	0.13

* KB-C-1 (cervical cancer) and SW-1271 (lung cancer) cell s are multidrug naïve, and KB-C2 (cervical cancer) and H69AR (lung cancer) are multidrug-resistant cancer cells.

Supplementary Table S2. A typical treatment protocol of engrafted mice with CTR alone or in combination with paclitaxel.

Treatment	Dosage	Frequency	Route	Notes
Sham control	Highest volume	Every 3-4 days	Intraperitoneal (I.P.)	Vehicle only
Tax[a]	10 mg/kg[b]	Once/week	Intravenous (I.V.)	
CTR-17 (30)	30 mg/kg	Every 3-4 days	I.P.	
CTR-20 (30)	30 mg/kg	Every 3-4 days	I.P.	

Tax (5), CTR-17 (15)	Tax, 5 mg & CTR17, 15 mg	Once/week	Tax (I.V.) & CTR-17 (I.P.)	Tax: given 24 h prior to CTR
Tax (5), CTR-20 (15)	Tax, 5 mg & CTR20, 15 mg	Once/week	Tax (I.V.) & CTR-20 (I.P.)	Tax: given 24 h prior to CTR

[a] Paclitaxel. [b] Kg body weight.

Supplementary Table S3: Antitumor activity of CTR-17 and CTR-20, alone or in combination with paclitaxel

	**Day 0**	**Day 6**	**Day 14**	**Day 17**	**Day 20**	**Day 24**	**Day 27**	**Day 30**
Sham control	89.61[c] ±8.97	105.03 ±15.90	136.66 ±16.87	162.62 ±15.29	192.69 ±16.56	268.08 ±37.85	426.87 ±7.57	557.66 ±24.72
Tax[a] (10 mg[b])	91.84 ±6.52	90.11 ±17.42	94.66 ±31.77	92.33 ±31.56	81.66 ±30.09	95.61 ±29.69	139.41 ±24.96	170.36 ±40.07
CTR-17 (30 mg)	86.71 ±5.42	63.99 ±3.85	90.07 ±21.30	128.36 ±20.65	134.76 ±26.46	160.86 ±37.30	189.45 ±47.61	209.84 ±56.45
CTR-20 (30 mg)	92.51 ±10.45	69.89 ±8.22	84.64 ±5.09	97.11 ±8.59	97.22 ±13.40	106.26 ±26.05	124.88 ±34.85	140.63 ±38.00
Tax (5 mg) plus CTR-17 (15 mg)	91.38 ±13.00	74.70 ±17.35	70.05 ±13.74	65.42 ±23.79	60.67 ±21.29	70.80 ±17.46	81.88 ±19.85	108.37 ±35.30
Tax (5 mg) plus CTR-20 (15 mg)	95.35 ±3.46	61.76 ±5.76	65.65 ±16.32	51.14 ±12.37	54.36 ±8.13	51.19 ±6.90	47.20 ±13.15	65.71 ±22.00

[a] Tax: paclitaxel. [b] mg per kg of body weight. [c] unit is mm^3.

Supplementary Figure legend

Supplementary Figure S1. CTR-20 effectively kill/inhibits the proliferation of cancer cells included in the NCI-60 panel. 10 μM of CTR-20 was used to examine the drug's efficacy against the NCI-60 cancer cell lines including: six leukemia cell lines, nine non-small cell lung cancer cell lines, seven colorectal cancer cell lines, six CNS cancer cell lines, nine melanoma cell lines, seven ovarian cancer cell lines, seven renal cancer cell lines, two prostate cancer cell lines and six breast cancer cell lines. The screening was carried out by the US National Cancer Institute (NCI) using a sulforhodamine B (SRB) colorimetric assay.

Supplementary Figures S2-S5. More detailed analysis of the CTR-20 effectiveness was carried out by the NCI using five different doses of CTR-20.

Supplementary Figure S6. CTR-17 caused apoptosis in a cancer-specific manner. Western blot analysis was carried out with an anti-PARP antibody at indicated time points using whole cell extracts prepared from asynchronous HeLa cancer or 184B5 non-cancer cells.

Supplementary Figure S7. Overexpression of MDR1 or MRP1 in the multidrug-resistant cells. (**a**) Western blotting was carried out with whole cell extracts prepared from either the parental KB-3-1 or MDR1-overexpressing KB-C-2 isogenic cell lines. (**b**) A similar experiment was carried out with extracts from the parental HEK293 and MRP1-overexpressing HEK293 isogenic cell lines. (**c**) Western blotting of MDA-MB-231 parental cells and paclitaxel-resistant MDA-MB-231TaxR cells.

Supplementary Figure S8. Number of mitotic cells increased in response to CTR-17 treatment. (**a**) Different cell types were either sham-treated or treated with 3.0 μmol/L of CTR-17 for 12 hours or 24 hours. The mitotic index was determined by fluorescence microscopy with counting at least 200 cells for each sample. The data are expressed as % of total cells, mean±S.E.M of at least two independent experiments. (**b**) The average distance between two centrosomes in the cell treated with CTR-17 (3.0 μmol/L) is shorter by ~35%, compared to the sham control. At least 100 cells, triplicate, were analyzed by fluorescence microscopy. The data were expressed as percentage mean±S.E.M of at least three independent experiments. (Related to Fig. 4)

Supplementary Figure S9. HeLa cells were arrested at the prometaphase-metaphase transition in response to CTR-17. (**a**) The prolonged arrest of cell cycle at or post-G2/M by CTR-17 eventually led to cell death without undergoing normal cell division. HeLa S3 cells synchronised at the G1/S border by double thymidine (DT) block were released into cell cycle in the absence (Sham control) or presence of 3.0 μmol/L CTR-17 at time 0 hour. “Async” denotes asynchronous cells. (**b**) Treatment of HeLa cells with 3.0 μmol/L of CTR-17 resulted in cell cycle arrest at prometaphase-metaphase transition. Western blotting was carried out with whole cell extracts prepared from HeLa cells that had been arrested at G1/S by DT treatment and subsequently released into complete medium in the absence (sham) or presence of 3.0 μmol/L of CTR-17. Equal amounts of protein samples were resolved by SDS-PAGE, followed by immunostaining

with antibodies specific for proteins listed on the left of the panel. GAPDH was used as the loading control. “p-“ denotes phosphorylation.

Supplementary Figure S10. CTR-17 caused prolonged spindle checkpoint activation. (**a**) Data from co-immunoprecipitation revealed that BubR1 was association with Cdc20, indicating that APC/C was as yet inactive, leading to prolonged spindle assembly checkpoint (SAC) activation. HeLa cells synchronised at the G1/S boundary by DT block were released into medium containing 50 ng/ml nocodazole or 3.0 μmol/L CTR-17, and then harvested at the indicated time points. BubR1 co-immunoprecipitates were resolved by SDS-PAGE and blotted with antibodies specific for proteins indicated. WCE denotes whole cell extract. (**b**) BubR1 is accumulated at the kinetochores in the cells treated with 3.0 μmol/L CTR-17 for 12 hours. HeLa cells, which had been either sham treated or treated with CTR-17 for 12 hours, were fixed and immunostained with antibodies specific for BubR1 or CENP (to visualize centromeres).

Supplementary Figure S11. CTR-17 and CTR-20 decreased the polymerized pool of tubulin. (**a**) HeLa, MDA-MB-231 and MDA-MB-468 cells were sham-treated, treated with 50.0 nmol/L of paclitaxel (Tax), 50.0 ng/ml of nocodazole (Noc), 3.0 μmol/L of CTR-17, or 1.0 μmol/L of CTR-20 for 12 hours. Cell lysates were separated into polymer (Pol) and soluble (Sol) fractions, and equal amounts of proteins were resolved by SDS-PAGE, followed by immunostaining with an antibody specific for α-tubulin. (**b**) The intensities of protein bands shown in panel **a** (HeLa) are shown in a graph format after they were scanned and quantitated. (Related to Fig. 5a)

Supplementary Figure S12. CTR-17 bound to tubulin through the colchicine-binding pocket. (**a**) CTR-17 quenched the intrinsic tryptophan fluorescence of tubulin in a dose-dependent manner. The changes in fluorescence intensity (ΔF) were plotted against the concentrations of compounds to determine the dissociation constant. Data are an average of five independent experiments (also see Fig. 5b). (**b**) CTR-17 (and CTR-20; see Fig. 5c) inhibited the binding of colchicine to tubulin. CTR-17 depressed the fluorescence of the colchicine-tubulin complex in a dose-dependent manner. The fluorescence intensity of the final tubulin complex was used to determine the inhibitory concentration (Ki) using a modified Dixon plot. The fluorescence intensity was normalized by subtracting any intrinsic fluorescence of CTR-17 at given doses

from that of the complex. “F” is the fluorescence of the CTR-colchicine-tubulin complex, and “F0” is the fluorescence of the colchicine-tubulin complex. Data are an average of at least four independent experiments. (**c**) Shown is the comparison of the chemical structures of colchicine, CTR-17, CTR-20 and podopillotoxin, all of which bind to the colchicine-binding pocket on the β-tubulin. (Related to Fig. 5)

Supplementary Figure S13. The binding sites for CTR-17 and CTR-20 on β-tubulin largely overlap with that of colchicine. Interactions between tubulin heterodimer (PDB code: 1SA0) and colchicine (**a**), CTR-20 (**b**), and CTR-17 (**c**) are shown in a 3D pattern (top panels). 2D ligand interaction diagrams show the bonds and amino acids within a distance of 4 Å to colchicine (**a**’), CTR-20 (**b**’) and CTR-17 (**c**’). There are three hydrogen (H) bonds between tubulin and colchicine, and two and one H bonds between tubulin and CTR-20 and CTR-17, respectively. Several hydrophobic and polar residues of the three compounds (colchicine, CTR-17 and CTR-20) interact with tubulin in a similar fashion. Amino acids which are common to colchicine and CTR-20 are shown in red boxes; those common to colchicine and CTR-17 are in yellow boxes; and those common to CTR-17 and CTR-20 are in blue boxes. (Related to Fig. 5d)

Supplementary Figure S14. The effects of CTR-17 and CTR-20 are reversible. (**a**) Flow cytometry profiles shown are for HeLa cells untreated, sham-treated, or treated with CTR-17 (3.0 μmol/L) or CTR-20 (1.0 μmol/L) for 12 hours, which is defined as time 0 hour (upper panels). The cells treated with CTR-17 or CTR-20 for 12 hours were washed twice with 1× PBS, and were then incubated for 3-12 hours in drug-free medium prior to analyzing cell cycle progression by flow cytometry (lower panels). (**b**) Samples of cell images at the indicated time points described in panel **a**.

Supplementary Figure S15. CTR-17 and CTR-20 do not show notable toxicity as measured changes in body and organ weights. (**a**) ATH490 mice were treated with paclitaxel (Tax), CTR compounds or in combination for 30 days. The body weights were normalized with those at day 0 (100%). The numbers in brackets are mg/kg body weight. (**b**) No significant difference was observed in the mass of four vital organs (liver, spleen, kidney, and lung of CD1 mice) between the vehicle control and drug-treated groups, as p values for the four organs were 0.42, 0.40, 0.55,

and 0.66, respectively. Organ weights were measured at 30-day post-treatment. Analysis was carried out using GraphPad Prism software. All values are presented as mean±S.E.M. Comparison between each group was made by p values determined using one-way ANOVA. The p value of <0.05 is considered to be statistically significant. Each organ weight was normalized with total body mass (BM) as expressed in %.

Supplementary Figure S16. Neither CTR-17 nor CTR-20 caused any notable toxicity to vital organs. ATH490 athymic mice were vehicle-treated (Sham) or treated with indicated compounds and doses for 30 days, followed by harvesting liver (and other organs; Fig. 7d) for analysis of their toxic effects. (**a**) Liver tissues stained with H & E. (**b**) Blood sera were collected from mice treated with indicated compounds and doses, and then examined the levels of ALT and AST to gain insight into liver toxicity. (**c**) Both CTR-17 and CTR-20 did not cause any notable side effects on vital mouse organs. The liver, spleen and kidney tissues maintained normal physiology in CD1 mice treated with CTR-17, CTR-20, or in combination with paclitaxel (Tax). The bar in the picture shows the expanded area of periarterial lymphatic sheaths in the spleen white pulp (WP) by the paclitaxel (10 mg/kg) treatment. The organs were harvested in the 30th day post-treatment. The tissue samples were fixed, processed, paraffin blocked, cut and H&E stained. H&E stained sections of liver, spleen and kidney were pictured at 20×, 10× and 40×, respectively, with a Zeiss EPI-fluorescent microscope. Higher than 10 layers in the marginal zone of mature B cells is considered the extension of WP, meaning the existence of high levels of B lympocites. The marginal zone of mature B cells and sometime integrated with some macrophages and dendritic cells. It should be noted that T cells are not observed because the animals used are athymic nude mice. All values are presented as mean±S.E.M.

Developmental Therapeutics Program

One Dose Mean Graph

NSC: D-793099 / 1 | **Conc:** 1.00E-5 Molar | **Test Date:** Sep 06, 2016

Experiment ID: 1609OS81 | **Report Date:** Sep 22, 2016

Panel/Cell Line	Growth Percent
**Leukemia**	
CCRF-CEM	7.53
HL-60(TB)	-2.46
K-562	4.24
MOLT-4	10.88
RPMI-8226	5.61
SR	2.69
**Non-Small Cell Lung Cancer**	
A549/ATCC	10.42
EKVX	37.76
HOP-62	8.67
HOP-92	-11.97
NCI-H226	38.93
NCI-H23	21.29
NCI-H322M	18.31
NCI-H460	1.79
NCI-H522	3.93
**Colon Cancer**	
COLO 205	20.45
HCC-2998	23.11
HCT-116	16.04
HCT-15	11.32
HT29	7.17
KM12	4.37
SW-620	8.40
**CNS Cancer**	
SF-268	9.36
SF-295	8.29
SF-539	-10.00
SNB-19	17.04
SNB-75	-11.48
U251	5.14
**Melanoma**	
LOX IMVI	10.23
MALME-3M	38.55
M14	13.82
MDA-MB-435	-10.05
SK-MEL-2	-2.52
SK-MEL-28	50.45
SK-MEL-5	25.21
UACC-257	37.62
UACC-62	25.73
**Ovarian Cancer**	
IGROV1	14.55
OVCAR-3	8.25
OVCAR-4	50.80
OVCAR-5	12.07
OVCAR-8	1.18
NCI/ADR-RES	10.36
SK-OV-3	6.12
**Renal Cancer**	
786-0	8.21
A498	21.71
ACHN	38.84
RXF 393	-7.90
SN12C	8.57
TK-10	42.01
UO-31	23.62
**Prostate Cancer**	
PC-3	22.06
DU-145	10.76
**Breast Cancer**	
MCF7	10.02
MDA-MB-231/ATCC	0.32
HS 578T	8.33
BT-549	18.91
T-47D	-2.02
MDA-MB-468	16.65
Mean	13.21
Delta	25.18
Range	62.77

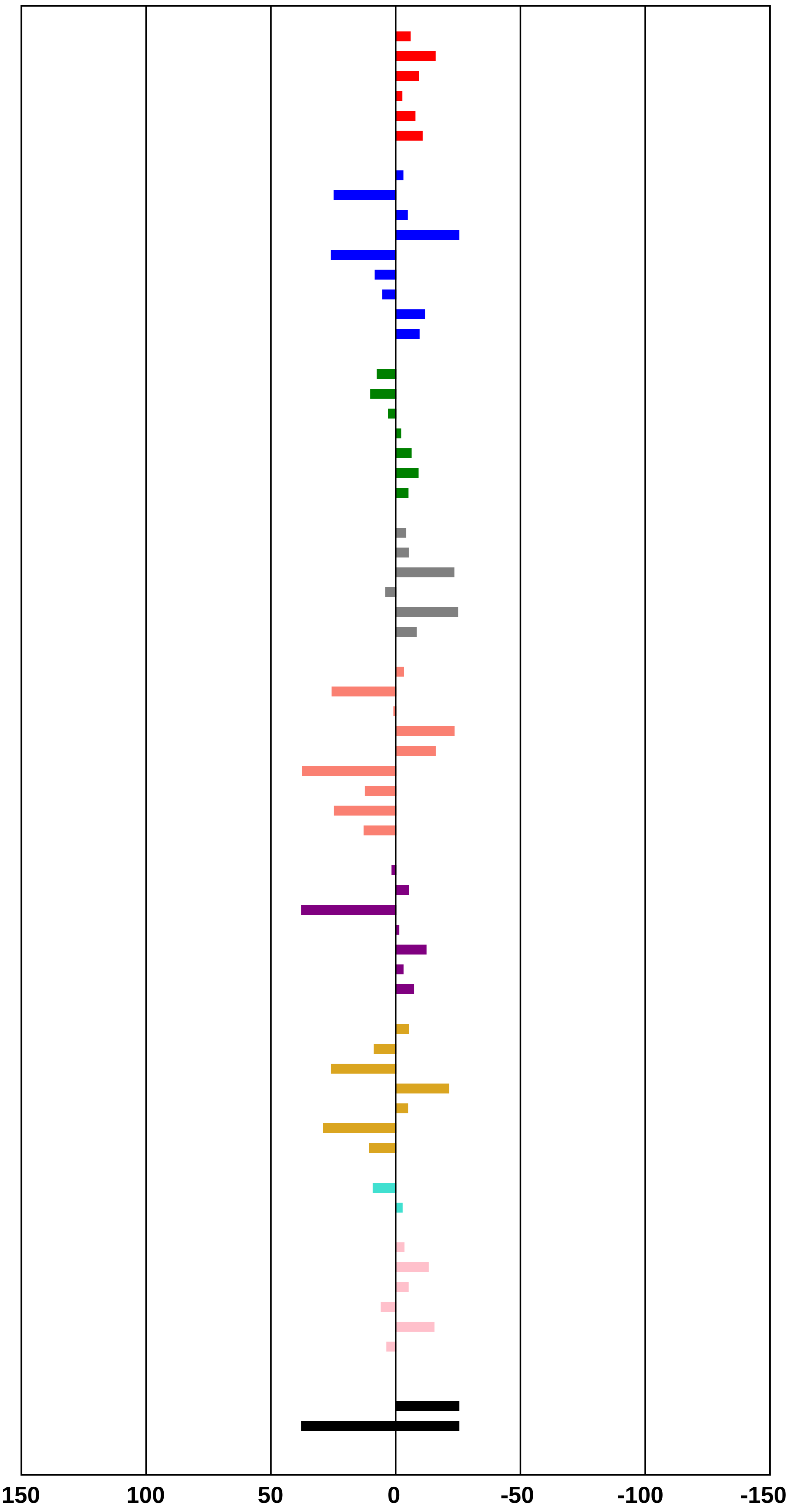

Supplementary Fig. S1

National Cancer Institute Developmental Therapeutics Program
Dose Response Curves

NSC: D - 793099 / 1	SSPL: 0ZTS	EXP. ID: 1610NS91
Report Date: January 18, 2017	Test Date: October 03, 2016	

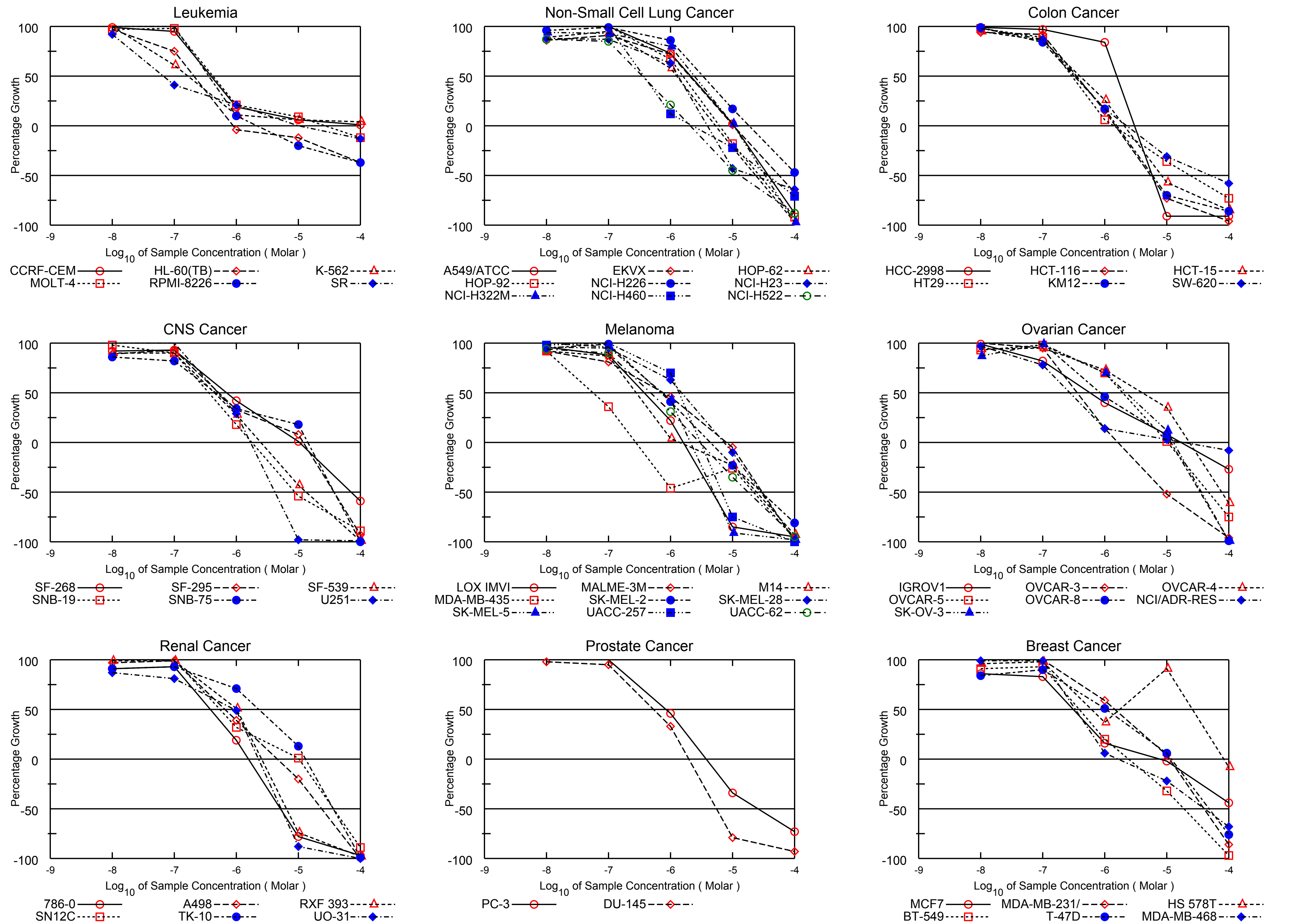

Supplementary Fig. S2

National Cancer Institute Developmental Therapeutics Program
In-Vitro Testing Results

NSC : D - 793099 / 1	Experiment ID : 1610NS91	Test Type : 08	Units : Molar
Report Date : January 18, 2017	Test Date : October 03, 2016	QNS :	MC :
COMI : CTR20	Stain Reagent : SRB Dual-Pass Related	SSPL : 0ZTS	

			Log10 Concentration												
	Time		Mean Optical Densities					Percent Growth							
Panel/Cell Line	Zero	Ctrl	-8.0	-7.0	-6.0	-5.0	-4.0	-8.0	-7.0	-6.0	-5.0	-4.0	GI50	TGI	LC50
Leukemia															
CCRF-CEM	0.438	1.340	1.331	1.291	0.614	0.489	0.447	99	95	19	6	1	3.92E-7	> 1.00E-4	> 1.00E-4
HL-60(TB)	0.596	1.888	1.851	1.564	0.571	0.523	0.376	97	75	-4	-12	-37	2.06E-7	8.83E-7	> 1.00E-4
K-562	0.248	1.630	1.640	1.084	0.396	0.327	0.303	101	61	11	6	4	1.62E-7	> 1.00E-4	> 1.00E-4
MOLT-4	0.467	1.720	1.687	1.699	0.730	0.584	0.410	97	98	21	9	-12	4.21E-7	2.71E-5	> 1.00E-4
RPMI-8226	0.570	1.949	2.122	2.031	0.713	0.458	0.360	113	106	10	-20	-37	3.85E-7	2.21E-6	> 1.00E-4
SR	0.376	1.505	1.412	0.835	0.610	0.378	0.328	92	41	21	.	-13	6.56E-8	1.03E-5	> 1.00E-4
Non-Small Cell Lung Cancer															
A549/ATCC	0.592	2.083	2.168	2.293	1.677	0.616	0.074	106	114	73	2	-88	2.09E-6	1.04E-5	3.79E-5
EKVX	1.071	2.670	2.452	2.530	2.223	1.094	0.349	86	91	72	1	-67	2.05E-6	1.05E-5	5.58E-5
HOP-62	0.630	1.358	1.281	1.321	1.049	0.493	0.018	89	95	58	-22	-97	1.24E-6	5.32E-6	2.37E-5
HOP-92	1.182	1.519	1.544	1.556	1.406	0.973	0.089	107	111	67	-18	-92	1.57E-6	6.17E-6	2.70E-5
NCI-H226	1.543	2.825	2.767	2.811	2.649	1.760	0.823	96	99	86	17	-47	3.34E-6	1.85E-5	> 1.00E-4
NCI-H23	0.781	2.054	1.900	1.889	1.585	0.444	0.285	88	87	63	-43	-64	1.33E-6	3.93E-6	2.16E-5
NCI-H322M	0.827	2.123	2.045	2.027	1.859	0.851	0.025	94	93	80	2	-97	2.40E-6	1.04E-5	3.35E-5
NCI-H460	0.333	2.860	2.999	2.914	0.641	0.259	0.096	105	102	12	-22	-71	3.80E-7	2.25E-6	3.67E-5
NCI-H522	0.994	2.240	2.082	2.055	1.253	0.544	0.119	87	85	21	-45	-88	3.52E-7	2.06E-6	1.29E-5
Colon Cancer															
HCC-2998	1.363	3.285	3.270	3.223	2.982	0.126	0.126	99	97	84	-91	-91	1.57E-6	3.03E-6	5.85E-6
HCT-116	0.296	1.922	1.822	1.784	0.539	0.080	0.013	94	92	15	-73	-96	3.48E-7	1.48E-6	5.48E-6
HCT-15	0.380	1.948	1.871	1.727	0.784	0.165	0.058	95	86	26	-57	-85	3.95E-7	2.05E-6	8.29E-6
HT29	0.310	1.653	1.628	1.501	0.388	0.198	0.084	98	89	6	-36	-73	2.93E-7	1.37E-6	2.37E-5
KM12	0.378	2.074	2.058	1.794	0.673	0.113	0.052	99	84	17	-70	-86	3.21E-7	1.58E-6	5.89E-6
SW-620	0.277	1.781	1.748	1.581	0.531	0.191	0.116	98	87	17	-31	-58	3.35E-7	2.24E-6	4.94E-5
CNS Cancer															
SF-268	0.542	1.823	1.718	1.738	1.076	0.560	0.221	92	93	42	1	-59	6.90E-7	1.05E-5	7.04E-5
SF-295	0.874	2.766	2.561	2.642	1.507	1.031	0.061	89	93	33	8	-93	5.29E-7	1.21E-5	3.76E-5
SF-539	1.105	2.923	2.747	2.740	1.618	0.631	0.014	90	90	28	-43	-99	4.43E-7	2.49E-6	1.34E-5
SNB-19	0.339	1.213	1.196	1.129	0.497	0.157	0.038	98	90	18	-54	-89	3.62E-7	1.79E-6	8.88E-6
SNB-75	0.890	1.829	1.699	1.660	1.213	1.059	0.003	86	82	34	18	-100	4.70E-7	1.42E-5	3.78E-5
U251	0.413	1.719	1.740	1.758	0.793	0.010	0.004	102	103	29	-98	-99	5.21E-7	1.70E-6	4.21E-6
Melanoma															
LOX IMVI	0.425	2.646	2.562	2.380	0.920	0.064	0.021	96	88	22	-85	-95	3.79E-7	1.61E-6	4.71E-6
MALME-3M	0.743	1.169	1.137	1.090	0.939	0.709	0.010	92	81	46	-5	-99	7.66E-7	8.09E-6	3.03E-5
M14	0.499	1.750	1.650	1.582	0.551	0.383	0.033	92	87	4	-23	-93	2.78E-7	1.41E-6	2.40E-5
MDA-MB-435	0.468	2.299	2.159	1.127	0.254	0.349	0.012	92	36	-46	-26	-97	5.64E-8	2.75E-7	2.19E-5
SK-MEL-2	0.919	1.897	1.845	1.883	1.318	0.711	0.171	95	99	41	-23	-81	6.94E-7	4.39E-6	2.92E-5
SK-MEL-28	0.738	1.946	1.899	1.885	1.504	0.665	0.016	96	95	63	-10	-98	1.52E-6	7.32E-6	2.85E-5
SK-MEL-5	0.751	2.813	2.830	2.736	1.630	0.067	0.019	101	96	43	-91	-98	7.28E-7	2.08E-6	4.93E-6
UACC-257	1.041	1.944	1.928	1.956	1.677	0.262	0.004	98	101	70	-75	-100	1.38E-6	3.05E-6	6.75E-6
UACC-62	0.969	2.988	2.869	2.782	1.599	0.632	0.050	94	90	31	-35	-95	4.78E-7	2.97E-6	1.79E-5
Ovarian Cancer															
IGROV1	0.538	1.870	1.859	1.632	1.073	0.644	0.394	99	82	40	8	-27	5.82E-7	1.70E-5	> 1.00E-4
OVCAR-3	0.429	1.650	1.673	1.584	0.603	0.206	0.018	102	95	14	-52	-96	3.59E-7	1.64E-6	9.33E-6
OVCAR-4	0.772	1.573	1.523	1.532	1.359	1.053	0.302	94	95	73	35	-61	4.07E-6	2.32E-5	7.69E-5
OVCAR-5	0.737	2.022	1.932	2.001	1.640	0.755	0.181	93	98	70	1	-75	1.97E-6	1.04E-5	4.66E-5
OVCAR-8	0.459	1.754	1.928	1.875	1.059	0.548	0.005	113	109	46	7	-99	8.74E-7	1.16E-5	3.44E-5
NCI/ADR-RES	0.571	1.784	1.748	1.519	0.736	0.611	0.528	97	78	14	3	-8	2.73E-7	2.00E-5	> 1.00E-4
SK-OV-3	0.864	1.978	1.830	1.962	1.648	0.996	0.009	87	99	70	12	-99	2.23E-6	1.28E-5	3.62E-5
Renal Cancer															
786-0	0.651	2.059	1.928	1.956	0.915	0.141	0.022	91	93	19	-78	-97	3.78E-7	1.56E-6	5.11E-6
A498	1.517	2.114	2.095	2.110	1.750	1.210	0.002	97	99	39	-20	-100	6.56E-7	4.55E-6	2.36E-5
RXF 393	0.985	1.583	1.577	1.580	1.291	0.261	0.020	99	99	51	-74	-98	1.02E-6	2.57E-6	6.48E-6
SN12C	0.429	1.655	1.676	1.651	0.817	0.443	0.046	102	100	32	1	-89	5.37E-7	1.03E-5	3.67E-5
TK-10	0.886	1.712	1.641	1.656	1.474	0.994	0.007	91	93	71	13	-99	2.31E-6	1.31E-5	3.64E-5
UO-31	0.669	2.015	1.838	1.754	1.334	0.078	-0.001	87	81	49	-88	-100	9.58E-7	2.28E-6	5.27E-6
Prostate Cancer															
PC-3	0.576	1.784	1.838	1.810	1.129	0.381	0.157	104	102	46	-34	-73	8.42E-7	3.75E-6	2.59E-5
DU-145	0.280	1.228	1.209	1.185	0.589	0.058	0.020	98	95	33	-79	-93	5.29E-7	1.96E-6	5.47E-6
Breast Cancer															
MCF7	0.413	2.070	1.836	1.792	0.678	0.404	0.230	86	83	16	-2	-44	3.12E-7	7.48E-6	> 1.00E-4
MDA-MB-231/ATCC	0.660	1.406	1.425	1.423	1.098	0.688	0.091	102	102	59	4	-86	1.44E-6	1.10E-5	3.96E-5
HS 578T	1.339	2.217	2.182	2.203	1.668	2.138	1.238	96	98	37	91	-8	.	8.38E-5	> 1.00E-4
BT-549	1.210	1.982	1.913	1.929	1.362	0.828	0.039	91	93	20	-32	-97	3.87E-7	2.42E-6	1.92E-5
T-47D	0.828	1.604	1.478	1.530	1.224	0.873	0.197	84	90	51	6	-76	1.06E-6	1.18E-5	4.79E-5
MDA-MB-468	0.834	1.806	1.796	1.793	0.895	0.647	0.266	99	99	6	-22	-68	3.36E-7	1.65E-6	4.01E-5

Supplementary Fig. S3

National Cancer Institute Developmental Therapeutics Program
Mean Graphs

NSC : D - 793099/1	Units :Molar	SSPL :0ZTS	EXP. ID :1610NS91
Report Date :January 18, 2017		Test Date :October 03, 2016	

Panel/Cell Line	Log_{10}GI50	Log_{10}TGI	Log_{10}LC50
**Leukemia**			
CCRF-CEM	-6.41	> -4.00	> -4.00
HL-60(TB)	-6.69	-6.05	> -4.00
K-562	-6.79	> -4.00	> -4.00
MOLT-4	-6.38	-4.57	> -4.00
RPMI-8226	-6.41	-5.66	> -4.00
SR	-7.18	-4.99	> -4.00
**Non-Small Cell Lung Cancer**			
A549/ATCC	-5.68	-4.98	-4.42
EKVX	-5.69	-4.98	-4.25
HOP-62	-5.91	-5.27	-4.63
HOP-92	-5.80	-5.21	-4.57
NCI-H226	-5.48	-4.73	> -4.00
NCI-H23	-5.88	-5.41	-4.66
NCI-H322M	-5.62	-4.98	-4.48
NCI-H460	-6.42	-5.65	-4.44
NCI-H522	-6.45	-5.69	-4.89
**Colon Cancer**			
HCC-2998	-5.80	-5.52	-5.23
HCT-116	-6.46	-5.83	-5.26
HCT-15	-6.40	-5.69	-5.08
HT29	-6.53	-5.86	-4.63
KM12	-6.49	-5.80	-5.23
SW-620	-6.47	-5.65	-4.31
**CNS Cancer**			
SF-268	-6.16	-4.98	-4.15
SF-295	-6.28	-4.92	-4.42
SF-539	-6.35	-5.60	-4.87
SNB-19	-6.44	-5.75	-5.05
SNB-75	-6.33	-4.85	-4.42
U251	-6.28	-5.77	-5.38
**Melanoma**			
LOX IMVI	-6.42	-5.79	-5.33
MALME-3M	-6.12	-5.09	-4.52
M14	-6.56	-5.85	-4.62
MDA-MB-435	-7.25	-6.56	-4.66
SK-MEL-2	-6.16	-5.36	-4.54
SK-MEL-28	-5.82	-5.14	-4.54
SK-MEL-5	-6.14	-5.68	-5.31
UACC-257	-5.86	-5.52	-5.17
UACC-62	-6.32	-5.53	-4.75
**Ovarian Cancer**			
IGROV1	-6.24	-4.77	> -4.00
OVCAR-3	-6.44	-5.78	-5.03
OVCAR-4	-5.39	-4.63	-4.11
OVCAR-5	-5.71	-4.98	-4.33
OVCAR-8	-6.06	-4.94	-4.46
NCI/ADR-RES	-6.56	-4.70	> -4.00
SK-OV-3	-5.65	-4.89	-4.44
**Renal Cancer**			
786-0	-6.42	-5.81	-5.29
A498	-6.18	-5.34	-4.63
RXF 393	-5.99	-5.59	-5.19
SN12C	-6.27	-4.99	-4.44
TK-10	-5.64	-4.88	-4.44
UO-31	-6.02	-5.64	-5.28
**Prostate Cancer**			
PC-3	-6.07	-5.43	-4.59
DU-145	-6.28	-5.71	-5.26
**Breast Cancer**			
MCF7	-6.51	-5.13	> -4.00
MDA-MB-231/ATCC	-5.84	-4.96	-4.40
HS 578T		-4.08	> -4.00
BT-549	-6.41	-5.62	-4.72
T-47D	-5.98	-4.93	-4.32
MDA-MB-468	-6.47	-5.78	-4.40
_MID	-6.21	-5.29	-4.58
Delta	1.04	1.27	0.8
Range	1.86	2.56	1.38

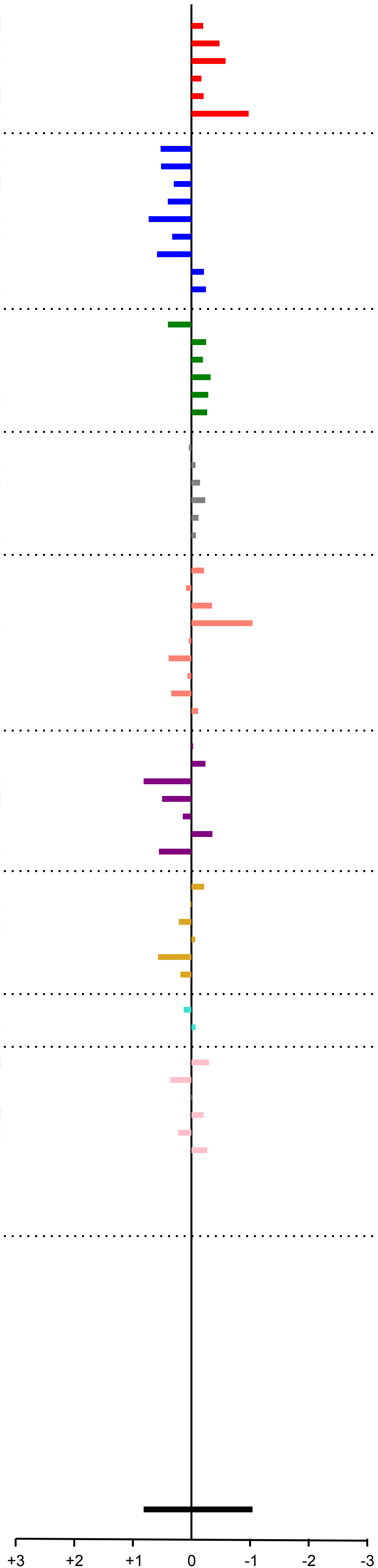

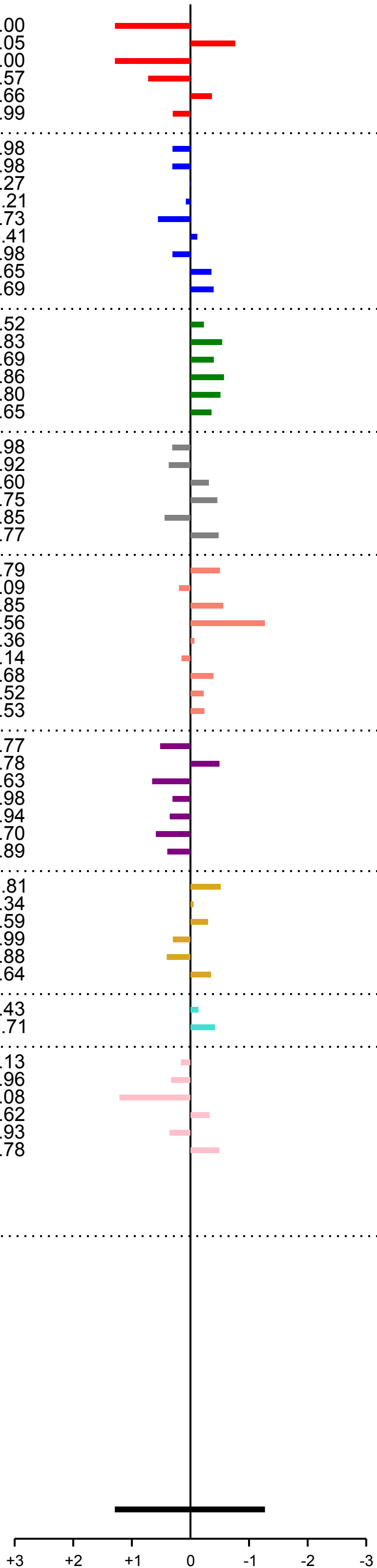

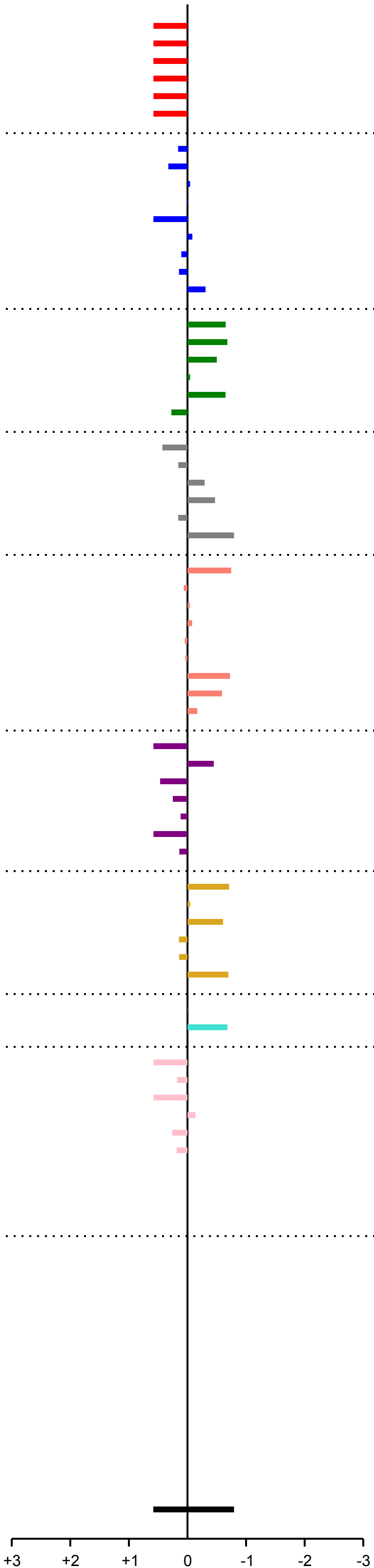

Supplementary Fig. S4

National Cancer Institute Developmental Therapeutics Program Dose Response Curves	NSC : D - 793099/1	SSPL:0ZTS	EXP. ID:1610NS91
	Report Date:January 18, 2017	Test Date:October 03, 2016	

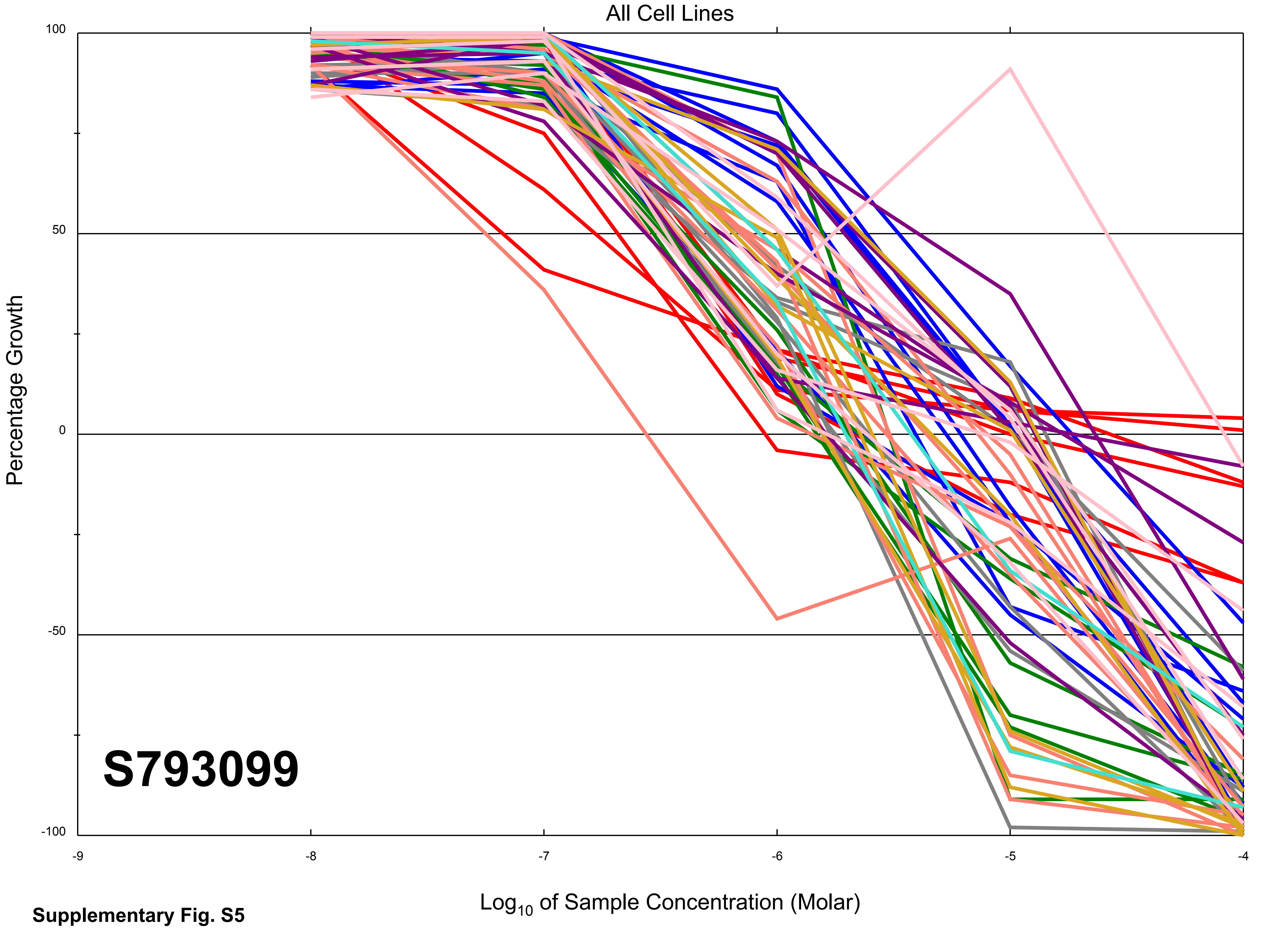

Supplementary Fig. S5

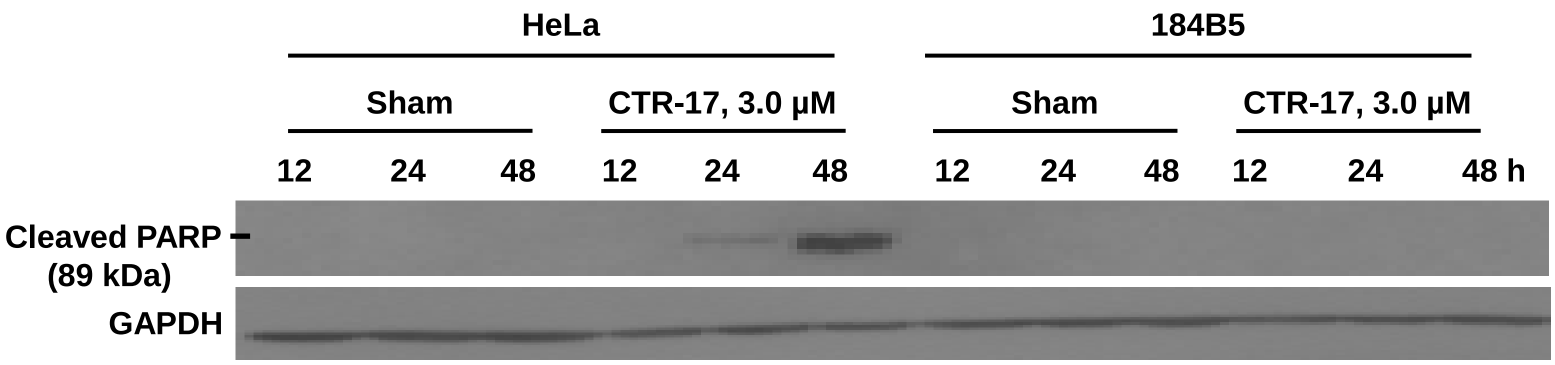

Supplementary Fig. S6

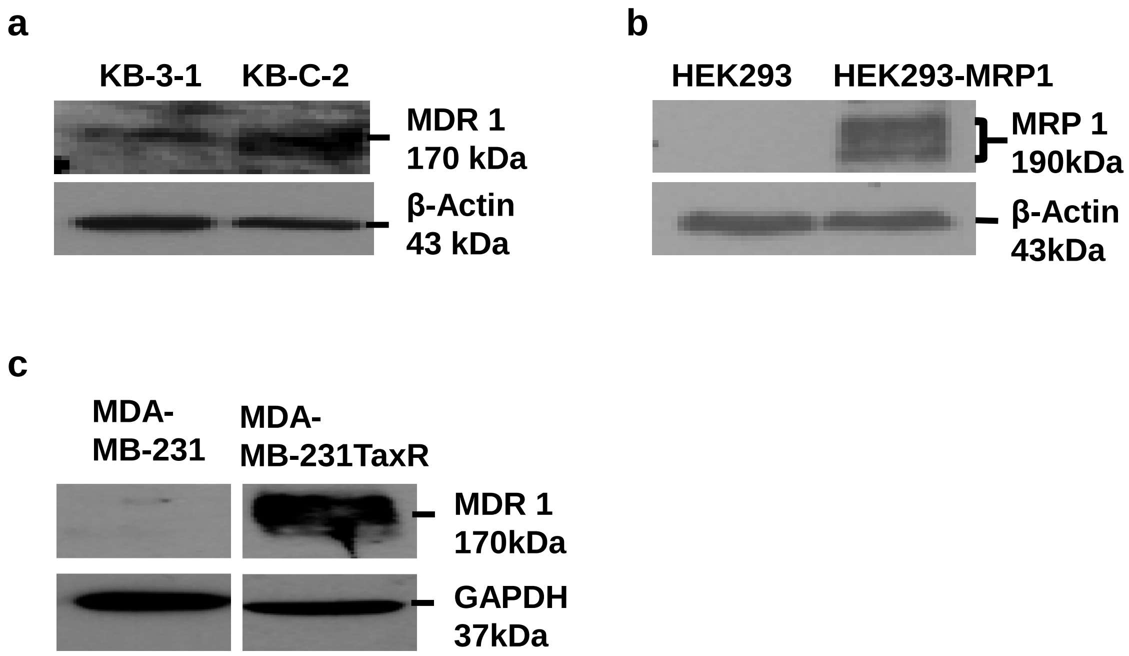

Supplementary Fig. S7

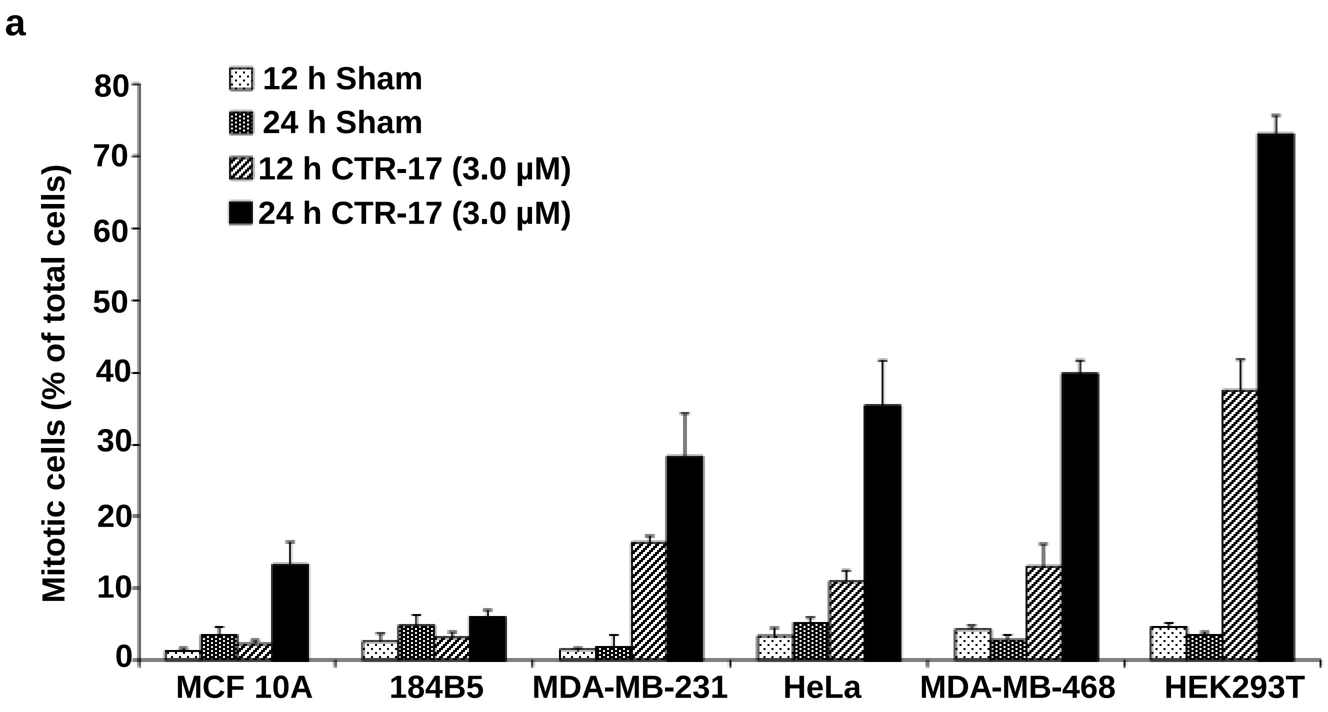

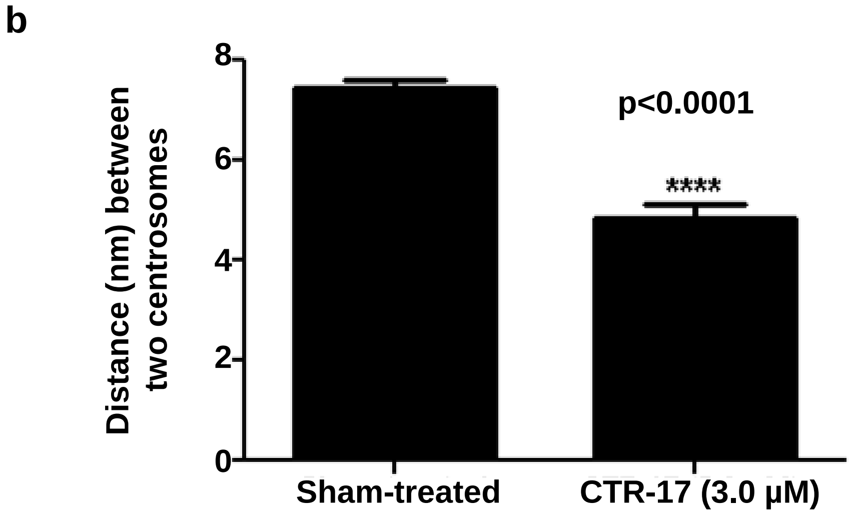

Supplementary Fig. S8

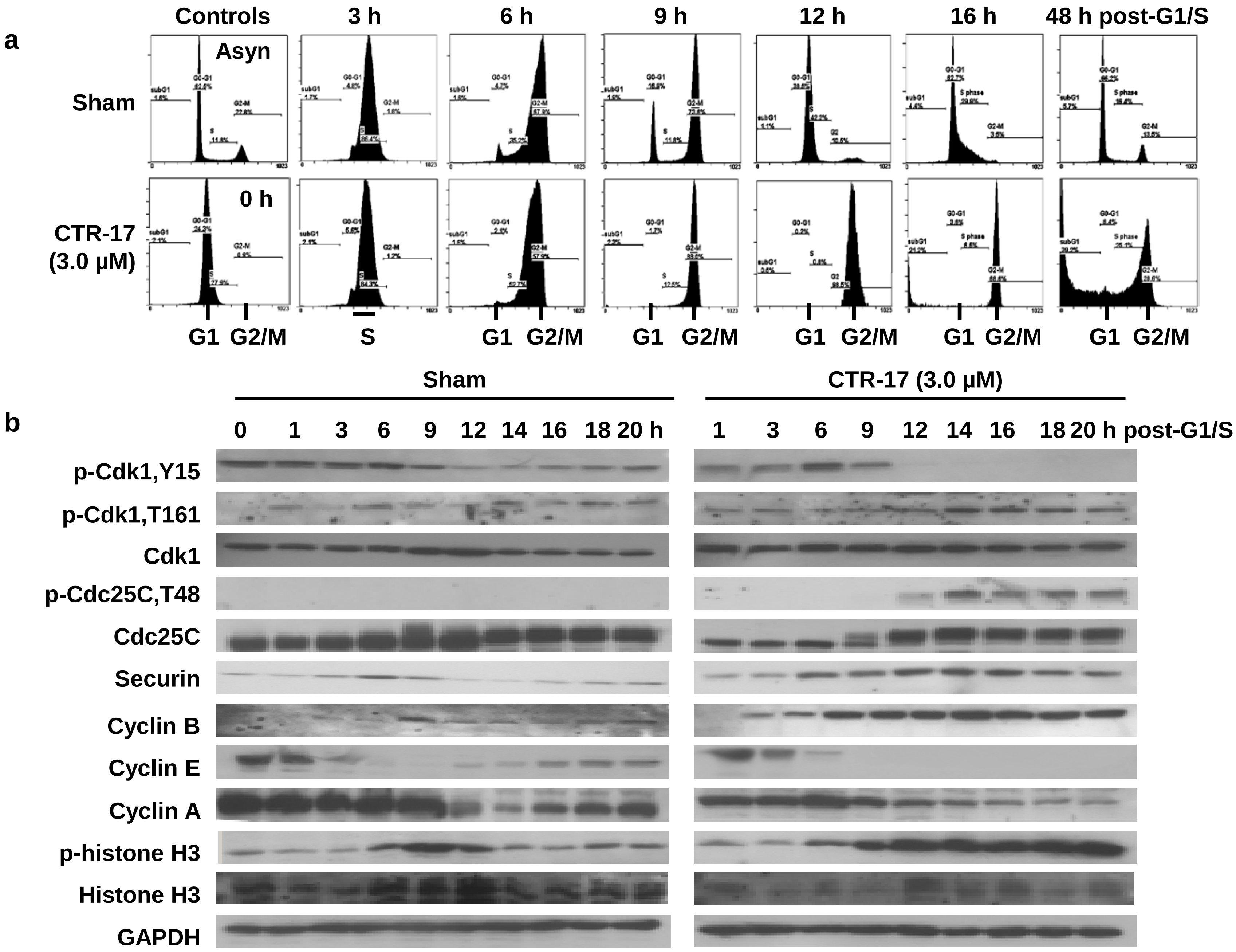

Supplementary Fig.. S9

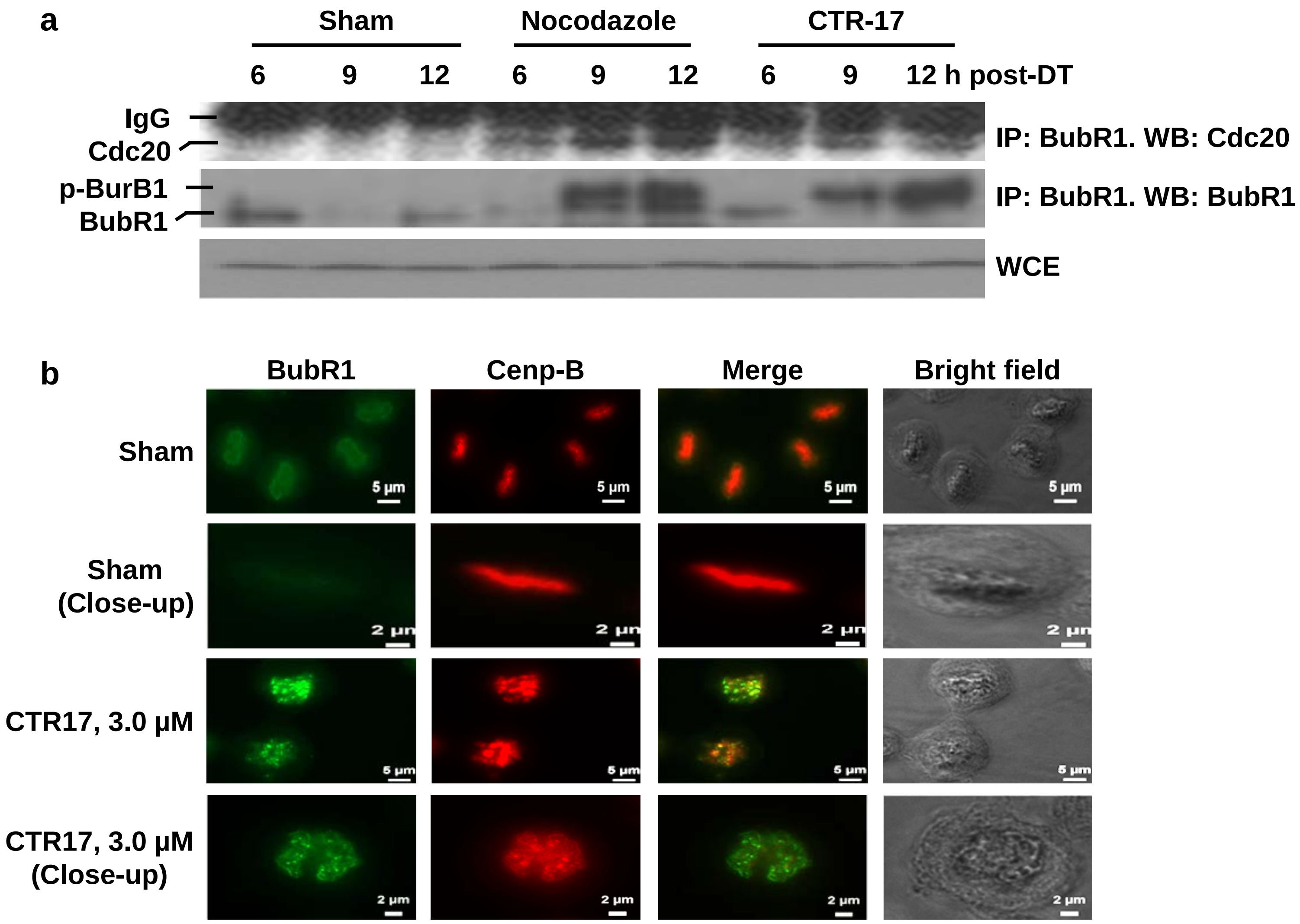

Supplementary Fig. S10

a

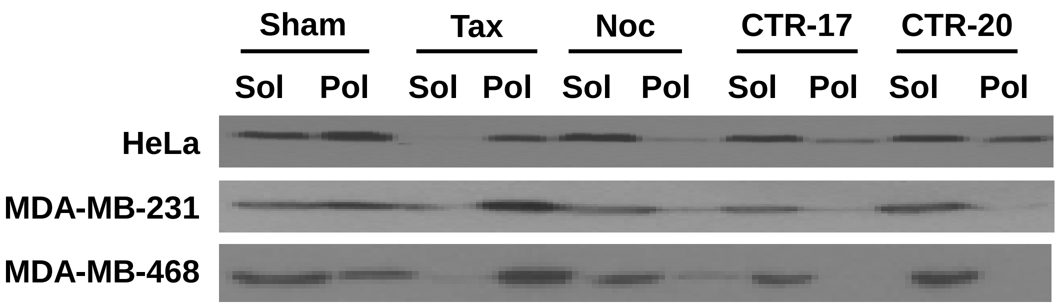

b

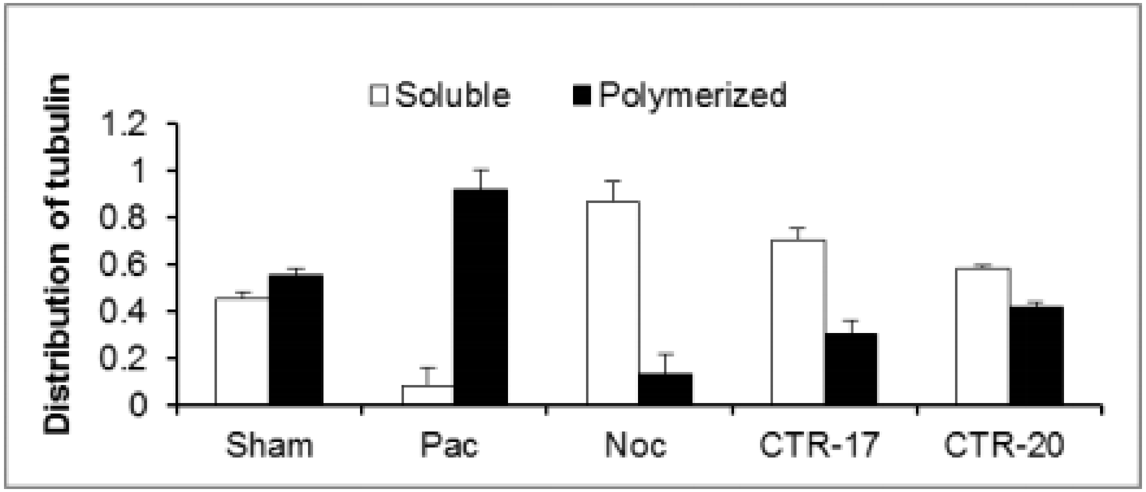

Supplementary Fig. S11

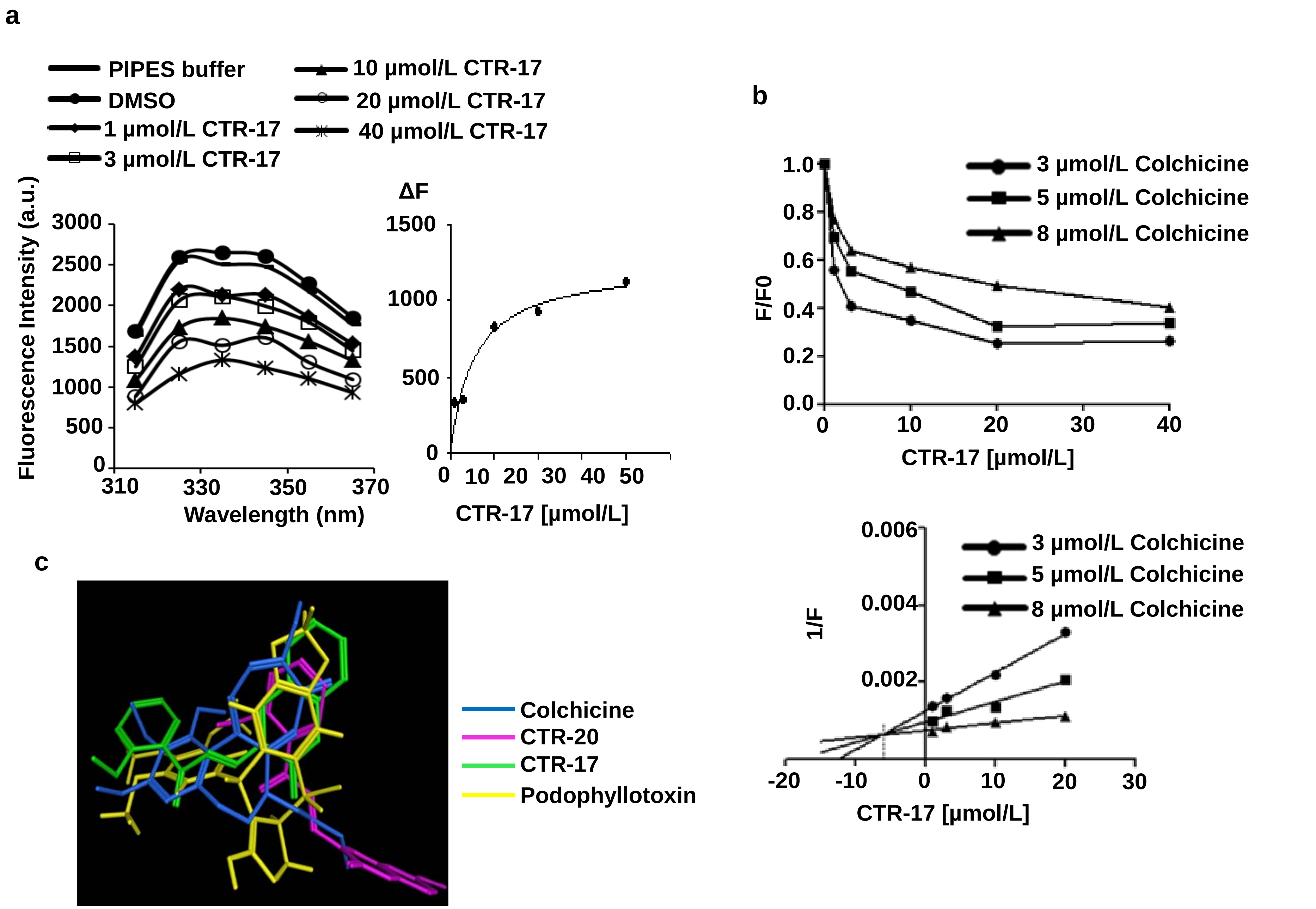

Supplementary Fig. S12

Colchicine bound to tubulin **CTR-20 bound to tubulin** **CTR-17 bound to tubulin**

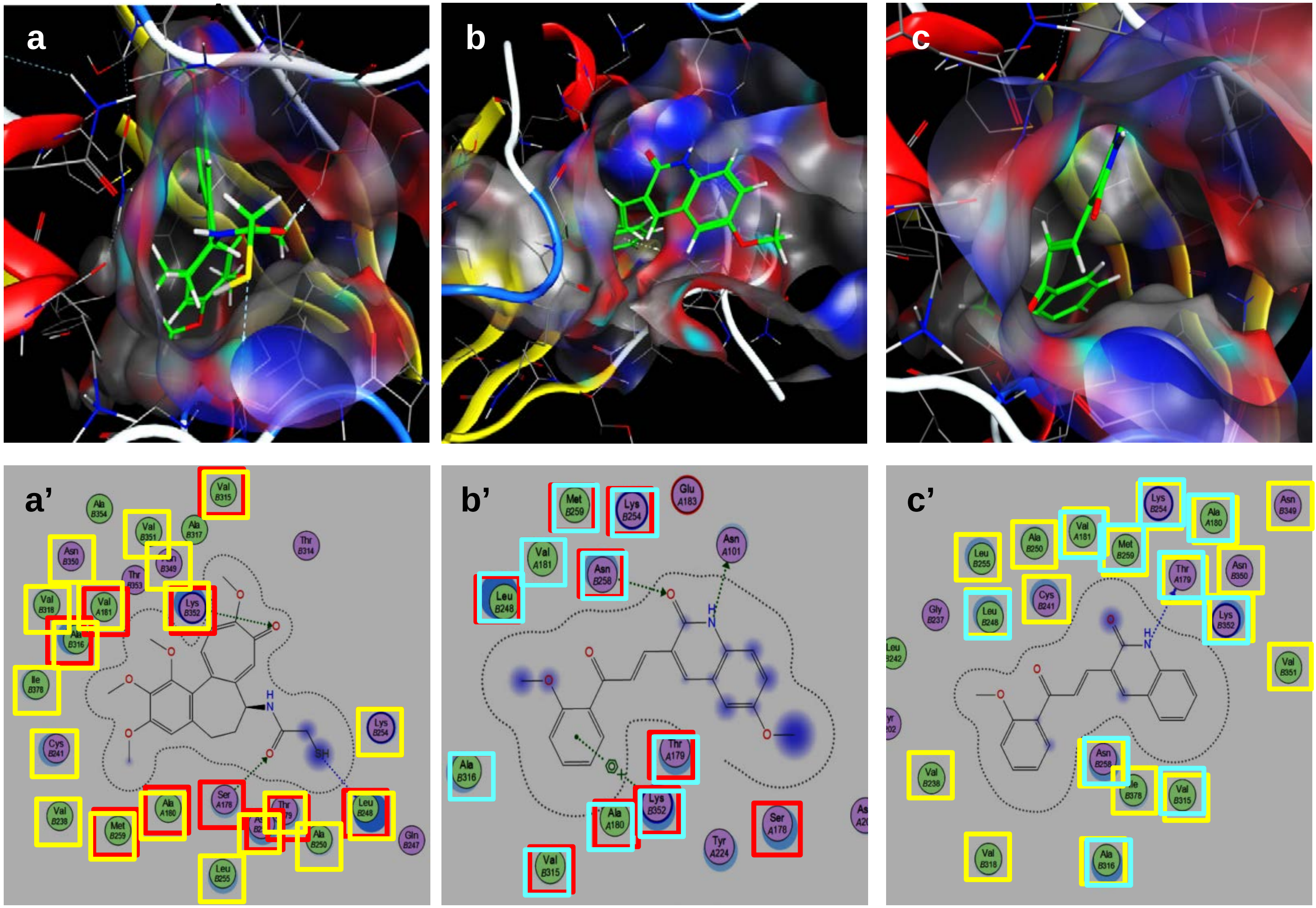

Supplementary Fig. S13

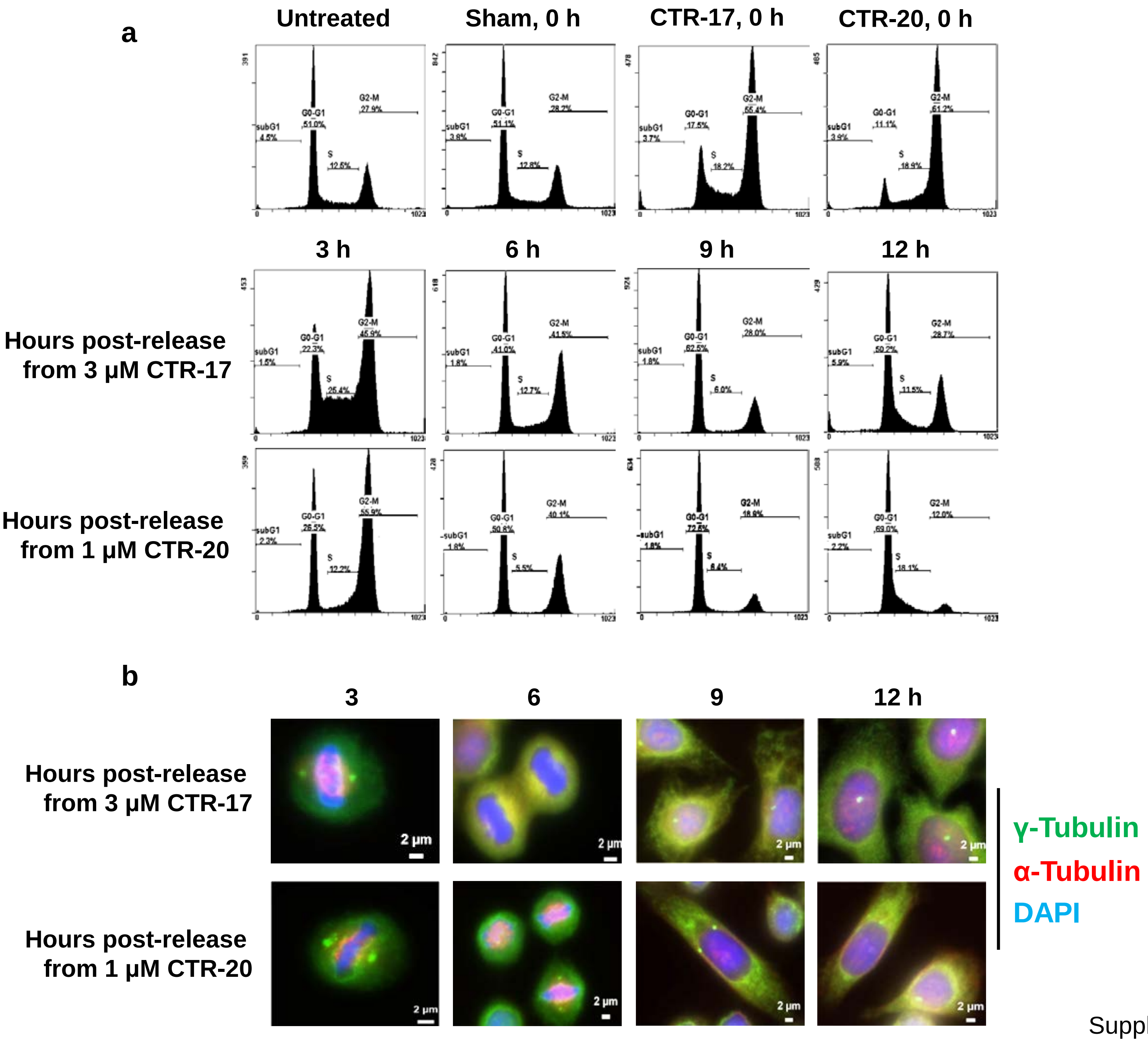

Supplementary Fig. S14

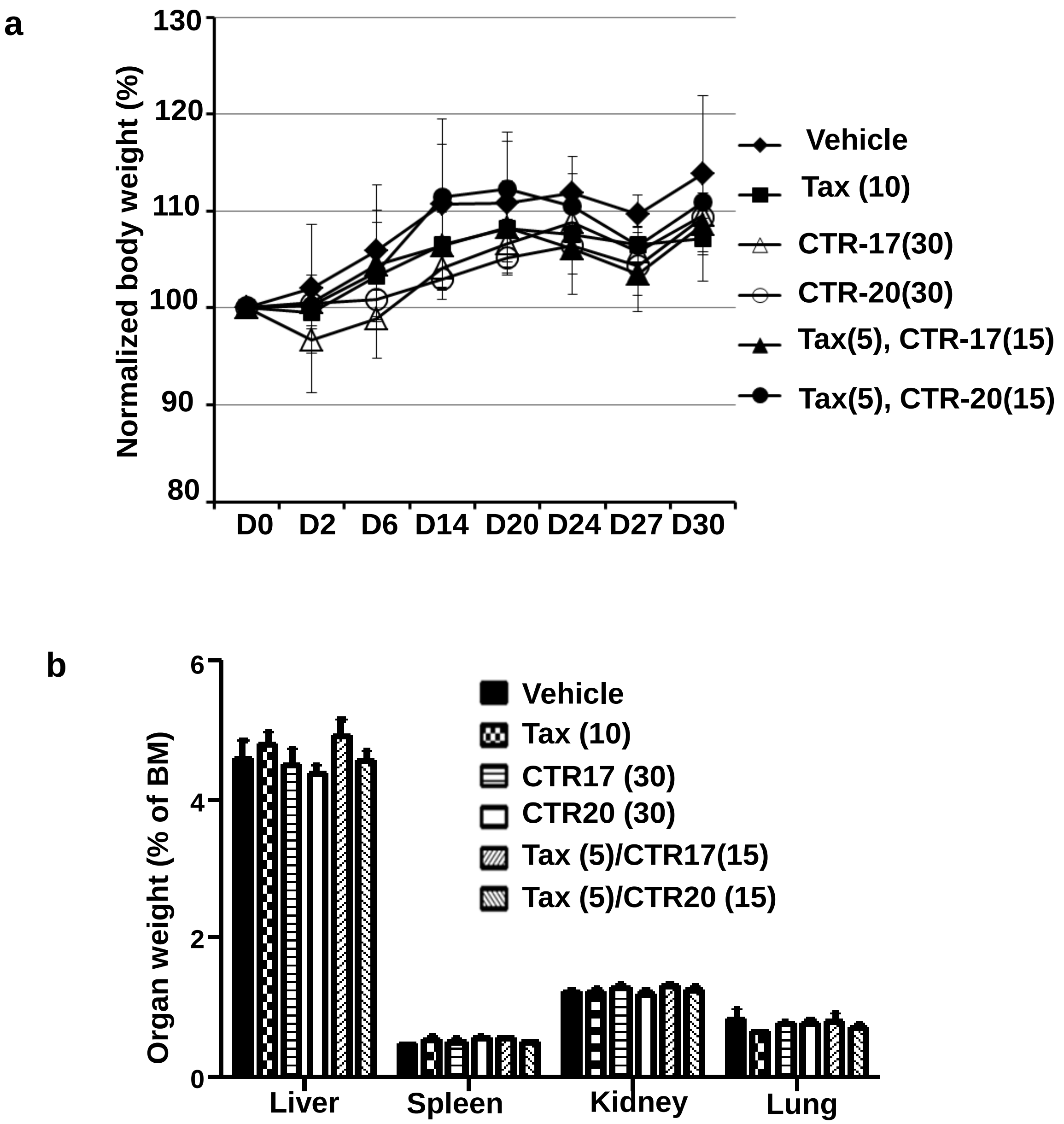

Supplementary Fig. S15

a

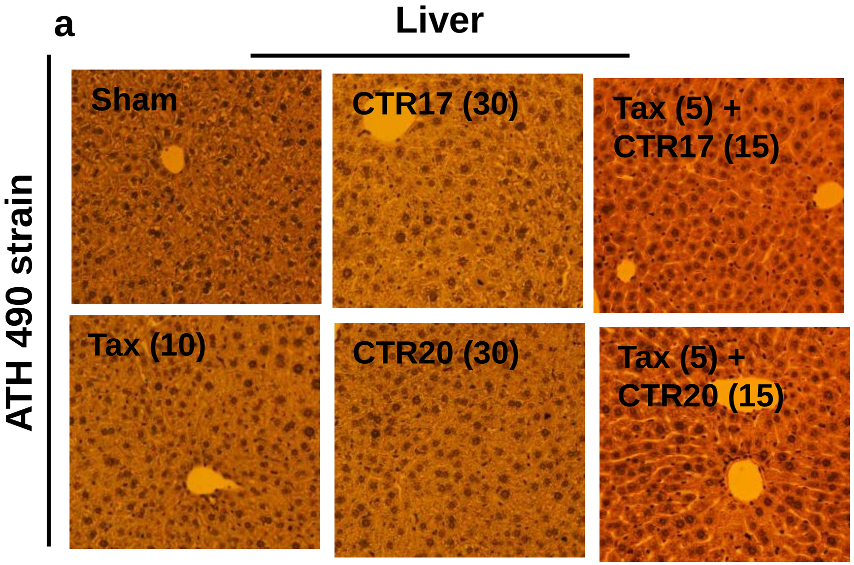

b

Treatment	ALT[a] (IU/L)	AST[b](IU/L)
Untreated	54.08±4.98	113.08± 9.13
Sham Control	64.03±4.04	112.46 ± 4.74
Paclitaxel (10 mg/kg)	52.02±2.59	111.14±13.42
CTR-17, 30 mg/kg	64.67±5.26	114.13 ± 6.36
CTR-20, 30 mg/kg	50.50±9.96	118.22±17.00
Paclitaxel (5), CTR-17 (15)	46.01±0.42	106.70±13.41
Paclitaxel (5), CTR-20 (15)	59.62±7.25	107.67±13.22

[a] ALT: Alanine transaminase.
[b] AST: aspartate aminotransferase.

c

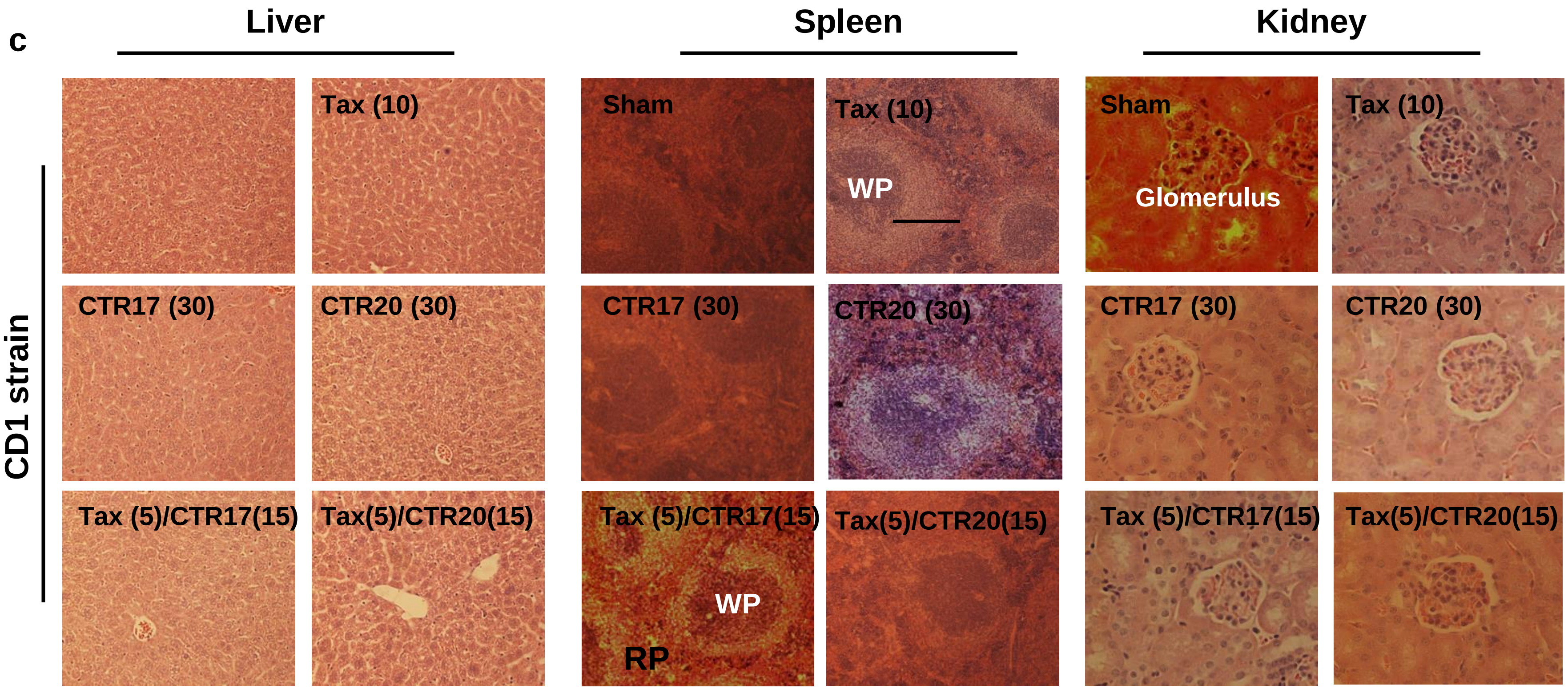

Supplementary Fig. S16
